# Specific and non-specific interactions of ParB with DNA: implications for chromosome segregation

**DOI:** 10.1093/nar/gku1295

**Published:** 2015-01-08

**Authors:** James A. Taylor, Cesar L. Pastrana, Annika Butterer, Christian Pernstich, Emma J. Gwynn, Frank Sobott, Fernando Moreno-Herrero, Mark S. Dillingham

**Affiliations:** 1 DNA:Protein Interactions Unit, School of Biochemistry, Medical Sciences Building, University of Bristol, Bristol, BS8 1TD, UK; 2 Department of Macromolecular Structures, Centro Nacional de Biotecnología, Consejo Superior de Investigaciones Científicas, 28049 Cantoblanco, Madrid, Spain; 3 Biomolecular & Analytical Mass Spectrometry Group, Department of Chemistry, University of Antwerp, Groenenborgerlaan 171, 2020 Antwerpen, Belgium; 4 Center for Proteomics (CFP-CeProMa), University of Antwerp, 2020 Antwerpen, Belgium

## Abstract

The segregation of many bacterial chromosomes is dependent on the interactions of ParB proteins with centromere-like DNA sequences called *parS* that are located close to the origin of replication. In this work, we have investigated the binding of *Bacillus subtilis* ParB to DNA *in vitro* using a variety of biochemical and biophysical techniques. We observe tight and specific binding of a ParB homodimer to the *parS* sequence. Binding of ParB to non-specific DNA is more complex and displays apparent positive co-operativity that is associated with the formation of larger, poorly defined, nucleoprotein complexes. Experiments with magnetic tweezers demonstrate that non-specific binding leads to DNA condensation that is reversible by protein unbinding or force. The condensed DNA structure is not well ordered and we infer that it is formed by many looping interactions between neighbouring DNA segments. Consistent with this view, ParB is also able to stabilize writhe in single supercoiled DNA molecules and to bridge segments from two different DNA molecules *in trans*. The experiments provide no evidence for the promotion of non-specific DNA binding and/or condensation events by the presence of *parS* sequences. The implications of these observations for chromosome segregation are discussed.

## INTRODUCTION

ParABS systems were discovered and defined as the DNA segregation apparatus for low copy number plasmids (1). They comprise the ParB protein, which binds specifically to the centromere-like DNA sequence *parS*, as well as ParA; a DNA binding protein and ATPase that binds DNA non-specifically in the presence of adenosine triphosphate (ATP). These three partitioning factors act together to distribute plasmids into daughter cells. A homologous apparatus is important for chromosome segregation during cell division or sporulation in many bacteria including *Bacillus subtilis* (2–8). Moreover, interactions between *B. subtilis* ParB (also called Spo0J) and *parS* are implicated in the recruitment of the Structural Maintenance of Chromosomes (SMC) complex (prokaryotic condensin) to the chromosome, providing an intriguing structural link between chromosome partitioning and condensation (9,10). In *B. subtilis, parS* is a 16-bp palindromic but partially degenerate DNA locus with the optimal sequence 5′-TGTTTCACGTGAAACA (11). Eight functional *parS* sequences are found in the origin proximal region of the *B. subtilis* chromosome (6) and ParB has been shown to associate with DNA for several kilobases around each of these sites (11,12). This phenomenon, which has been referred to as ‘spreading’ to form ‘ParB domains’, has been shown to be important for function in some cases (11–13) but does not appear to be a universal feature of ParB proteins, which are diverse both at the level of structure and biochemical function and have consequently been grouped into different types (14). The mechanism of ParB spreading from *parS*, along with many other aspects of chromosomal ParABS function, is not fully understood.

Crystal structures of ParB proteins from plasmid and genomic partitioning systems have provided important insights into their modes of DNA binding. The *B. subtilis* ParB protein studied here is a member of the Type Ia centromere binding protein family (14) and for this class of proteins, structural information is available for a *Thermus thermophilus* orthologue, as well as for the more distantly related plasmid-encoded systems P1 ParB, KorB and SopB (15–20). Taken as a whole and together with other studies (21,22), these structures reveal a shared general architecture consisting of three functional regions: an N-terminal domain that is important for interaction with ParA, a central region containing a helix-turn-helix motif needed for specific DNA binding to *parS*, and a C-terminal dimerization domain which, in at least some cases, acts as a second DNA binding locus. However, the details of how these proteins assemble to form higher order ‘segrosome’ complexes with their DNA targets remain to be determined and may differ, even between ParB proteins that are related at the level of primary structure (14).

Electrophoretic mobility shift assays (EMSAs) on short oligonucleotide substrates, as well as footprinting assays, have demonstrated specific binding of *parS* by *B. subtilis* ParB *in vitro*, but do not provide evidence for the lateral spreading that had been expected based on the *in vivo* behaviour (11,12). Moreover, several previous studies using EMSAs with longer DNA substrates showed an apparent ‘laddering’ effect that is consistent with non-specific coating of DNA by ParB, but did not directly address the role of *parS* sequences in (potentially) nucleating these structures (10,12). Recently, single molecule imaging studies have revealed that several different ParB proteins are able to condense flow-stretched DNA, and it has been suggested that this is the result of protein-mediated bridges between distant non-specific DNA segments (23). The possible role of *parS* in this activity was unclear because condensation was observed on substrates lacking specific sites, and ParB localization at *parS* was not detected. Based in part upon this work, modelling of ParB binding to DNA has suggested a ‘two-state nucleation model’ for ParB binding and localized condensation of DNA around *parS* (24). In this model, a combination of 3D bridging and lateral spreading protein–protein interactions forms a network of ParB protomers that together condense DNA, and it is proposed that such networks are most efficiently nucleated by *parS*-bound ParB molecules.

Using bulk biochemical assays, we show here that *B. subtilis* ParB binds to DNA both specifically and non-specifically. Specific binding involves a well-defined complex between a ParB homodimer and a single *parS* sequence, whereas non-specific binding is co-operative and associated with the formation of large and poorly defined structures. Importantly, these different modes of DNA binding appear largely independent of one another, and we find no evidence for the nucleation of the larger non-specific complexes by *parS* sequences. Single-molecule experiments with magnetic tweezers (MTs) show that ParB is capable of packaging non-specific DNA with a low condensing force of 2.1 pN. This condensation activity is reversible and due to the formation of loops between non-specific DNA segments that are bridged *in cis* by multiple ParB proteins. Bridging *in trans* was also observed in experiments involving multiple-tethered DNA molecules. The large nucleoprotein complexes are presumably formed by a combination of ParB–ParB interactions together with non-specific ParB–DNA interactions. We speculate that non-specific binding may occur at an unidentified site on the protein, such that the nucleoprotein condensates could be anchored around *parS* by an independent and highly specific binding locus associated with the helix-turn-helix motifs.

## MATERIALS AND METHODS

### DNA vector and substrate preparation

The *B. subtilis parB* gene, which contains a single *parS* site (the sequence in the coding strand is 5′-TGTTCCACGTGAAACA), was amplified from *B. subtilis* 168 genomic DNA and cloned into the vectors pET28a(+) (Novagen) and pSP73 (Promega). For the production of non-specific DNA substrates a series of point mutations were introduced into the *parS* site by site-directed mutagenesis of the pET28a-ParB and pSP73-ParB constructs using the QuikChange kit (Stratagene). These mutations preserved the GC content of the site, but render the site not competent for ParB binding. The sequence of this ‘scrambled’ *parS* site was as follows (mutations in bold): 5′-**C**GT**G**CC**CA**G**G**GA**G**ACA. All DNA substrates longer than 100 bp were produced by polymerase chain reaction (PCR) using pSP73 ParB as a template. Those smaller than 100 bp were produced by annealing 1:1 mixtures of complementary oligonucleotides (ATDBio or Eurofins MWG Operon) in 10-mM Tris·HCl pH 8.0, 50-mM NaCl, 1-mM ethylenediaminetetraacetic acid (EDTA) by heating to 95°C for 5 min followed by slow cooling to room temperature. Competitor DNA for MT assays was produced following the same procedure in 10-mM Tris-HCl pH 7.5, 100-mM NaCl, using complementary 50-bp *parS* or 50-bp non-specific oligonucleotides. Where appropriate, substrates were radiolabelled by 5′-end labelling with γ32P-ATP (Perkin Elmer) and polynucleotide kinase (NEB) according to manufacturer's instructions. All oligonucleotide sequences used in this study are listed in Supplementary Table S1. The *parS* sequence used in the oligonucleotide substrates is the same as that found in the *parB* gene and used in the PCR-based substrates.

Substrates for MT experiments consist of a large central part (∼6 kb) containing either a single *parS* or a non-specific scrambled *parS* site, as well as two smaller fragments (∼1 kb) labelled with biotins or digoxigenins. The central part was produced by PCR using a high-fidelity DNA polymerase (Phusion Polymerase, NEB) and the pET28a(+) ParB plasmid with existing NotI and XhoI sites having been removed by site-directed mutagenesis (QuikChange kit, Stratagene). The smaller fragments labelled with biotins or digoxigenins were PCR fabricated from plasmid pSP73-JY0 (25,26). PCR conditions were as described by the manufacturer and the products were checked by gel electrophoresis. The labelled fragments and the long central segment were cut with NotI and XhoI (NEB) and ligated overnight at 16°C with T4 DNA ligase (NEB) followed by heat inactivation for 20 min at 65°C. In summary, the standard DNA molecules used in MT experiments have an accessible length of 5994 bp flanked by two labelled ends (of 968 and 997 bp) that specifically bind either to a glass surface covered with Anti-DIG or to superparamagnetic beads covered with streptavidin. About 30% of molecules tested with the MT were torsionally constrained when fabricated using the procedure described above. DNAs were never exposed to intercalant dyes or UV radiation during their production and were stored at 4°C.

### Protein preparation

Untagged ParB protein was expressed and purified as follows. pET28a-ParB was transformed into BL21 (DE3) competent cells and transformants were used to inoculate 4 l of Luria Broth (LB) media containing kanamycin. Cells were grown at 37°C to an OD_600_ of 0.5, at which time 1-mM Isopropyl β-D-1-thiogalactopyranoside (IPTG) was added followed by a further 3-h incubation. Cells were harvested and resuspended in 50-mM Tris·HCl (pH 8.0), 500-mM NaCl, 1-mM EDTA, 1-mM DTT, 5% v/v glycerol and protease inhibitor cocktail set II (Millipore) before being stored at −80°C. Cells were thawed and lysed by 0.2-mg/ml lysozyme (Sigma) and sonication. The lysate was clarified by centrifugation at 50 000 x g for 30 min at 4°C. Ammonium sulphate was added to 40% saturation for 30 min at 4°C with stirring, before centrifugation at 10 000 xg at 4°C for 15 min. Ammonium sulphate was added to the resulting supernatant to increase its saturation to 50%, and the ParB protein was pelleted by centrifugation at 10 000 x g, 4°C for 15 min.

The ParB containing pellet was resuspended in 50-mM Tris·HCl pH 7.5, 1-mM EDTA, 2-mM β-mercaptoethanol to a final conductivity of 15 mS/cm. The protein was loaded on to a 5-ml HiTrap Heparin HP column (GE Healthcare) equilibrated with 50-mM Tris·HCl pH 7.5, 1-mM EDTA, 2-mM β-mercaptoethanol, 150-mM NaCl at 1.5 ml/min, and eluted with a gradient of 150-mM NaCl to 750-mM NaCl over 50 ml. ParB containing fractions were pooled and diluted to a conductivity of 15 mS/cm before being loaded onto 2x 1-ml Mono S columns (GE Healthcare) equilibrated with 50-mM Tris·HCl pH 7.5, 1-mM EDTA, 2-mM β-mercaptoethanol, 150-mM NaCl at 1 ml/min. ParB was eluted with a gradient from 150–750-mM NaCl over 20 ml. ParB containing fractions were then pooled and concentrated by centrifugation in Amicon Ultra-15 3 kDa cutoff spin filters (Millipore) before dialysis against 50-mM Tris·HCl pH 7.5, 1-mM EDTA, 1-mM DTT, 300-mM NaCl. Glycerol was added to a final concentration of 10% (v/v), the purified ParB protein was then aliquoted, snap frozen in liquid nitrogen and stored at −80°C. The protein concentration was determined before addition of glycerol by absorbance at 280 nm, using a theoretical extinction co-efficient of 7450 M^−1^ cm^−1^. Some ParB preparations were found to be contaminated with an unidentified nuclease and were additionally purified using a Hiload 16/600 Superdex S75 gel filtration column (GE Healthcare) equilibrated with 50-mM Tris·HCl pH 7.5, 1-mM EDTA, 1-mM DTT, 300-mM NaCl loaded at 0.5 ml/min. The protein was then concentrated and stored as above.

### Electrophoretic mobility shift assays

**Figure 1.**
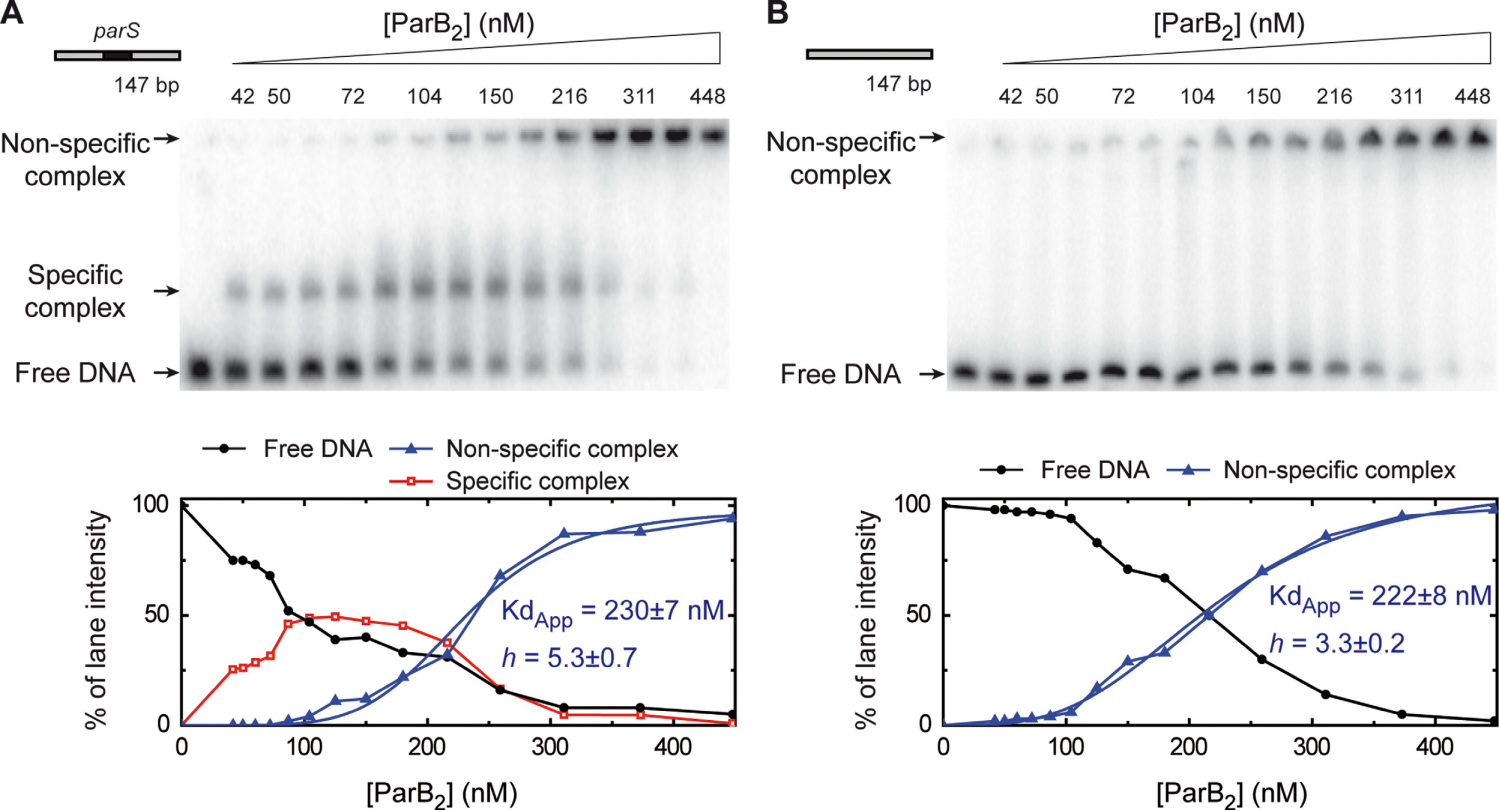
Specific binding of ParB to the *parS* sequence. Electrophoretic mobility shift assay of ParB binding to a radiolabelled 147-bp substrate in a magnesium acetate containing gel-running buffer. (**A**) Titration of ParB on DNA containing a single *parS* site in the centre. (**B**) ParB titration on an equivalent substrate that is lacking a *parS* site (see Supplementary Table S1 for details). The species assigned as specific and non-specific complexes are labelled. The lower panels show the quantification of the gels revealing a highly sigmoidal pattern for non-specific binding. These data were fit to Equation (1) to yield the values shown.

ParB was incubated with 20-nM 147-bp DNA substrate (consisting of 19-nM cold DNA spiked with 1-nM radiolabelled DNA), 50-mM Hepes·KOH pH 7.5, 100-mM KCl, 2.5-mM MgCl_2_, 0.1-mg/ml bovine serum albumin (BSA), 1-mM DTT and 2.5% (v/v) Ficoll in a 25-μl reaction volume. Samples were incubated at room temperature for 30 min followed by 5 min on ice. Complexes were resolved on 6% acrylamide/bis-acrylamide (29:1) gels in 90-mM Tris, 150-mM Boric acid (final pH 7.5). Gels were supplemented with either 2.5-mM Magnesium acetate (TBM gel) or 1-mM EDTA (TBE gel) depending on the experiment, and were pre-run at 150 V, 4°C for 30 min in a buffer identical to their composition. Ten microliters of each sample was loaded and the gels run at 150 V, 4°C for 1 h. The gels were then dried under vacuum and visualized by exposure to a phosphor screen, which was subsequently scanned by a phosphorimager (Typhoon FLA 9500, GE Healthcare). Gels were quantified using ImageQuant (GE Healthcare) and each band expressed as a percentage of the sum of all bands. In performing EMSA analyses, we initially used standard TBE running buffers but found that it was not possible to detect specific ParB–*parS* complexes under these conditions. We subsequently discovered that either TBM running buffer (see above) or 10-mM NaH_2_PO_4–_20 mM KOAc pH 6.5 running buffer did allow the detection of the specific complexes (see main text and Figure 1 and Supplementary Figure S1).

The non-specific DNA binding data were sigmoidal, suggesting positive co-operativity, and were treated semi-quantitatively by fitting to Equation (1),

(1) \begin{equation*} Y = \frac{{{\it B}_{{\rm max}} \times [{\rm ParB}_{\rm 2} ]^h }}{{Kd_{{\rm app}}^h + [{\rm ParB}_{\rm 2} ]^h }}, \end{equation*}

where *Y* is the measured binding signal, *B*_max_ is the maximum binding signal, *h* is the Hill coefficient and *Kd*_app_ is an apparent dissociation constant.

### DNA binding assays using fluorescence

For assays monitoring the fluorescence change of Cy3-labelled DNA upon binding to ParB, ParB (at the concentration indicated) was incubated with 20-nM 148-bp Cy3-labelled DNA substrate (see Supplementary Table S1) in a buffer containing 50-mM Hepes·KOH (pH 7.5), 100-mM KCl, 2.5-mM MgCl_2_, 0.1-mg/ml BSA and 1-mM DTT. Samples were analysed using a Cary Eclipse fluorimeter with excitation at 549 nm and an emission scan taken between 560 and 600 nm. The maximum peak height in this range was measured and the percentage increase in this value relative to a control experiment lacking ParB is reported. The data were fit to Equation (1). The errors reported are the standard error of three independent data sets.

### Limited proteolysis

ParB_2_ (2 μM) was incubated for 10 min at room temperature in the presence or absence of 20 μM of either 50-bp *parS*-containing or 50-bp non-specific DNA (see Supplementary Table S1). Trypsin (Sigma) was then added to a final concentration of 1 ng/μl and the samples incubated at room temperature. At the time intervals shown, 30 μl of each sample was removed and mixed with 2.5 μl 100-mM PMSF (Sigma) and 27.5 μl 2x Stop buffer (62.5-mM Tris·HCl pH 6.8, 25% v/v glycerol, 2% w/v sodium dodecyl sulphate (SDS), 5% v/v β-mercaptoethanol, 0.5% w/v bromophenol blue) to quench the reaction, followed by incubation at 95°C for 3 min. Samples were resolved by loading 15 μl on to a 15% discontinuous SDS-polyacrylamide gel electrophoresis (PAGE). The gel was then visualized using InstantBlue stain (Expedeon). Quantification was carried out in ImageJ (NIH) and the molecular weights of each species were compared to a Precision Plus Protein marker (Biorad). This analysis determined the molecular weight of intact monomeric ParB to be 31.6 kDa compared to the theoretical weight of 32.2 kDa, giving an approximate error of 0.6 kDa. Trypsin digest products were also analysed by N-terminal sequencing. The experiment was carried out as above, except the gel was left unstained and the digest products transferred to a polyvinylidene difluoride (PVDF) membrane. The membrane was then stained (0.1% w/v Coomassie Brilliant Blue, 40% v/v isopropanol, 10% v/v acetic acid) and the bands of interest excised. These products were analysed by N-terminal peptide sequencing (Alta Bioscience) which yielded amino acid sequences that matched ParB, and which were not found in trypsin (see main text for details).

### Size exclusion chromatography with multi-angle light scattering

ParB samples at either 9 or 80-μM ParB_2_ were incubated in the presence or absence of 10-μM Hex-labelled DNA in 50-mM Hepes·KOH pH 7.5, 150-mM KCl, 2.5-mM MgCl_2_ and 2-mM β-mercaptoethanol at room temperature for 30 min on ice. They were then run at 1 ml/min in 50-mM Hepes·KOH, 150-mM KCl, 2.5-mM MgCl_2_ and 2-mM β-mercaptoethanol on a Superdex 200 analytical size-exclusion column (GE Healthcare) attached to a light scattering diode array (Dawn Heleos II, Wyatt Technology, UK) and a differential refractive index detector (Optilab rEX, Wyatt Technology UK). This allows for the accurate measurement of a complexes molecular mass without bias from its shape by calculating the ratio of light scattering to the differential refractive index of a sample. Additionally, the detectors can measure the absorbance of a sample at a certain wavelength allowing the separate tracking of protein/DNA (absorbance 280 nm) or Hex-labelled DNA (absorbance 535 nm) as indicated in the figures. Chromatograms were analysed using the ASTRA software (v6.0.5.3, Wyatt Technology UK), and the weighted average mass of species determined along with the error in this calculation (typically less than 5%). The reproducibility of runs was assessed using a BSA standard before and after each experiment to see if there was variability in either calculated molecular weight or elution volume. No detectable change was observed over these experiments.

### Native mass spectrometry

ParB was incubated with 100-bp *parS* DNA in a 1:1 ratio (5-μM ParB_2_ and DNA) for 5 min at room temperature before buffer exchanging into 300-mM ammonium acetate (Sigma) using Micro Bio-Spin P-6 Gel columns (BioRad). For measurements in the absence of DNA, 5-μM ParB_2_ was directly buffer exchanged into 300-mM ammonium acetate. Measurements were performed on a Waters Synapt G2 HDMS T-wave ion mobility mass spectrometer with nano-electrospray using in-house-made gold-coated borosilicate capillaries. The following parameters were applied with the aim to preserve non-covalent interactions (27): backing pressure 4.3 mbar, source pressure 5.3 × 10^−3^ mbar, trap pressure 1.7 × 10^−2^ mbar; capillary voltage 1.7 kV, sampling cone 30 V, extraction cone 0.5 V, trap and transfer collision energy 25 V and 0 V, trap DC bias 10 V, source temperature 30°C.

### Magnetic-tweezers assays

We employed an MT setup similar to an apparatus that has been described previously (28,29). Characteristic features of this equipment, for example the range of accessible forces as well as the time and spatial resolution, have been reported previously (30). The DNA sample was mixed in phosphate buffered saline (PBS) with 1-μm diameter magnetic beads (Dynabeads, MyOne streptavidin, Invitrogen) that were previously washed three times in PBS and diluted 1:8 from the stock solution. The DNA/beads ratio was adjusted empirically to obtain single- or double-tethered beads that were identified by rotation of the magnets. DNA molecules and beads were incubated for 5 min and injected in the flow cell. Following a 10-min bead adsorption we applied a force of 4 pN to remove non-attached beads and flowed the reaction buffer consisting of 100-mM NaCl, 50-mM Hepes-KOH pH 7.5, 4-mM MgCl_2_, 1-mM DTT, 100-μg/ml BSA, 0.1% TWEEN 20. ParB_2_ protein was injected at 1 μM in the reaction buffer and the extension of the DNA recorded after 2-min incubation. Additional injections related to the flow of DNA competitor are indicated in some figures using a horizontal bar, the position and length of which indicate the starting time and the duration of the injection, respectively.

## RESULTS

### Specific and non-specific DNA binding modes of ParB

It has been shown previously that *B. subtilis* ParB is associated with non-specific DNA for several kilobases either side of its specific DNA binding sites *in vivo* (11,12). This has led to the hypothesis that the *parS* sequence acts as a nucleating site from which ParB spreads either laterally (12) or by the formation of loops (23). However, *in vitro* studies of *B. subtilis* ParB binding and spreading have produced contradictory and confusing results. For example, studies that used EMSAs to investigate binding to long DNAs produced a ladder of ParB:DNA complexes, apparently consistent with coating of non-specific DNA, whilst DNAse I footprinting showed only protection of *parS* with no evidence for spreading to non-specific sites (10,12). Furthermore recent studies on ParB binding to flow-stretched lambda DNA were unable to visualize binding of fluorescently labelled ParB to *parS*, but could observe rapid non-specific binding which resulted in DNA condensation (23).

To investigate binding of ParB to specific and non-specific DNA we first developed a protocol for the purification of native (untagged) ParB protein and then re-visited the use of EMSA assays using a variety of different buffer compositions for resolving nucleoprotein complexes in the gels. When TBE buffer was used for the resolving gel as in previous experiments (Supplementary Figure S1) (6,10,12), we found that ParB bound to a 147-bp DNA molecule to form a ladder of shifted bands that was highly reminiscent of those seen before (10,12). Importantly however, there was no quantitative or qualitative difference in the pattern of bands produced when the DNA either contained a single central *parS* sequence or a ‘scrambled’ non-specific control sequence (for details of the substrates see Supplementary Table S1). Therefore, under these conditions, not only is there no support for the idea that *parS* promotes spreading of ParB onto non-specific DNA, there is actually no evidence for the specific binding of *parS* sequences at all. This control has not been reported in some other studies of ParB binding to longer substrates, although ParB has been shown to bind preferentially to *parS* in EMSAs with oligonucleotides that are shorter than ∼30 bp (6,12). We subsequently found that, by replacing EDTA in the gel running buffer with magnesium acetate, we were able to clearly reveal the specific binding of ParB to *parS* on long DNA molecules (Figure 1 and Supplementary Figure S1). Under these conditions, the *parS* substrate shows a shift at low [ParB] to form a single well-defined complex that runs into the gel, and a supershift of this material to a position close to the wells of the gel at elevated [ParB]. When the control DNA is used, the well-resolved intermediate band is not observed, whereas the shift to the wells still occurs, and does so at concentrations of ParB similar to those for the *parS*-containing substrate. We interpret the intermediate band as a specific complex of ParB bound to the *parS* sequence and estimate that the dissociation constant for this interaction is on the order of 100 nM under these conditions. However, this value should be interpreted cautiously as EMSA is a quasi-equilibrium technique, and the presence of ParA proteins may also enhance specificity for *parS* in some cases (31). The non-specific binding of DNA that is observed at higher [ParB] might be accompanied by the formation of large nucleoprotein networks, given that these complexes are very poorly resolved by EMSA. This non-specific binding also appears to be cooperative as no intermediary species are observed and quantification of the gels reveals a highly sigmoidal pattern for the formation of this species (Figure 1 lower panels; this will be expanded upon below). The fact that we did not see a substantial effect of *parS* on the formation of this higher-order complex suggests that, although we can observe highly specific complexes of ParB at the *parS* site, such complexes do not appear to act as a recruitment site for further ParB binding to adjacent non-specific DNA under these conditions. Similar results were obtained with running buffers containing alternative divalent cations including calcium or manganese, or if the running buffer was altered to a pH 6.5 phosphate buffer (data not shown). The enhancement in specificity (compared to TBE gels) that we observe here might therefore relate to a general effect on the charge and/or conformation of the DNA that disfavours non-specific binding.

**Figure 2.**
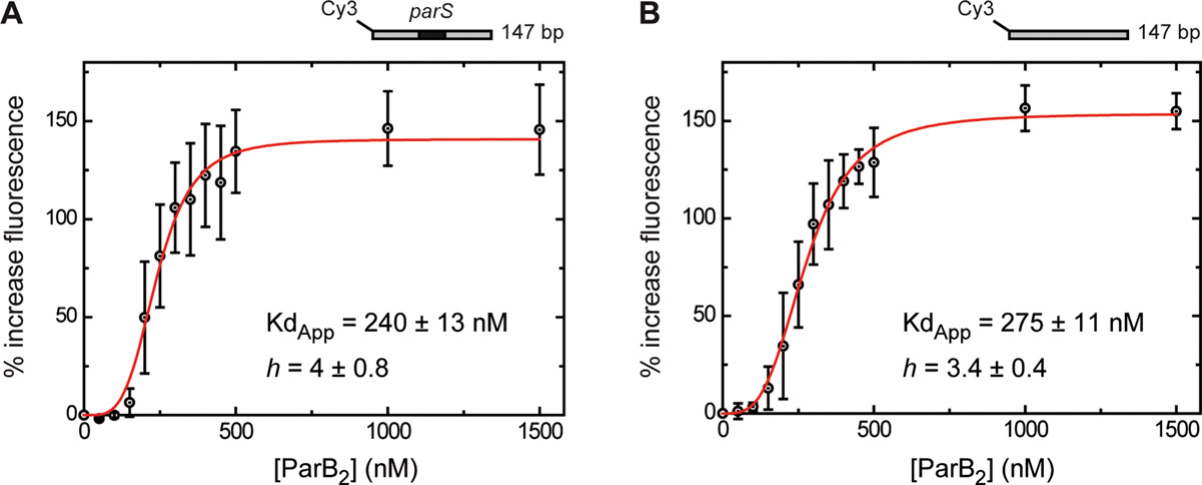
The ParB–*parS* complex does not recruit additional ParB molecules to neighbouring non-specific DNA. The binding of ParB to a 147-bp DNA labelled with Cy3 (Supplementary Table S1) results in an increase in fluorescence intensity. (**A**) Titration of ParB dimer on DNA containing a *parS* site in its centre. (**B**) Titration on an equivalent substrate that is lacking the *parS* site. These data were fit to Equation (1) to yield the values shown. The error bars represent the standard errors from three independent experiments.

To investigate the binding of ParB to DNA more quantitatively and without the complications of a gel-based resolution step, we sought to develop a solution-based assay for ParB binding. Binding of a protein adjacent to a fluorophore on a labelled piece of DNA can change its fluorescence properties (32), and so we designed 5′-Cy3-labelled DNA substrates that are otherwise equivalent to those used in the EMSAs. Since the *parS* site is positioned in the centre of these substrates, at a considerable distance from the fluorophore, we reasoned that this might allow us to detect recruitment of ParB to adjacent non-specific DNA by *parS*. Titration of ParB against these substrates resulted in a concentration-dependent increase in Cy3 fluorescence to about 2.5-fold at saturation (Figure 2). The binding isotherm was clearly sigmoidal, with virtually no binding signal observed below 100-nM ParB_2_, demonstrating a strong apparent positive co-operativity. The data were well described by a Hill equation (Equation (1)) and the apparent *Kd* and Hill coefficients were similar for both *parS-*containing (*Kd* = 240 ± 13 nM, *h* = 4 ± 0.8) and *parS*-free substrates (275 ± 11 nM, *h* = 3.4 ± 0.4). Given the very different nature of the assays, these values are in reasonable agreement with those measured for formation of the non-specific complexes by EMSA (compare graphs in Figures 1 and 2), albeit with a somewhat higher Hill co-efficient measured for the *parS* substrate with EMSA. Consequently, we conclude that this solution-based fluorescence assay is likely to be reporting on the formation of the same non-specific (and presumably large) nucleoprotein complexes that we observe near the wells of EMSA experiments. Importantly, we again find that the presence or absence of a single *parS* sequence does not significantly affect the binding of ParB to neighbouring non-specific DNA. The kinetics of the ParB-dependent fluorescence increase were slow and complex, requiring tens of seconds to reach equilibrium at high [ParB] and displaying a distinct lag phase (Supplementary Figure S2). The presence or absence of a *parS* sequence had no substantial effect on the kinetics of the fluorescence increase. Together, the data presented above show that ParB binds both specifically and non-specifically to DNA *in vitro*, leading to the formation of distinct complexes in native gels, and that these DNA binding modes are substantially independent.

### The relationship between ParB architecture and DNA binding mode

**Figure 3.**
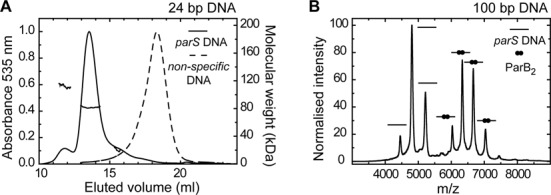
The stoichiometry of the ParB–*parS* complex. (**A**) Binding of ParB_2_ (9 μM) to 24-bp Hex-labelled DNA (10 μM) analysed by SEC-MALS. Only DNA-containing species were observed by monitoring the (normalized) absorbance at 535 nm. With a *parS* containing DNA substrate (solid line) the major complex has a calculated Mw of 81.6 ± 1.9 kDa, consistent with a single ParB dimer bound to DNA. A lower abundance species is also seen with a calculated Mw of 112.1 ± 3. In contrast, ParB is unable to bind a non-specific substrate (dotted line). In that case, the DNA is found in a late eluting peak, for which no weight could be assigned due to poor light scattering. (**B**) Native-mass spectrometry of ParB binding to a 100-bp substrate containing a single *parS* sequence predominantly showed a single dimer bound to the DNA, as well as free DNA. The peak assignments are indicated using cartoons on the graph. Binding of ParB to non-specific DNA was not observed.

Since our binding studies were unable to provide structural information on the specific and non-specific nucleoprotein complexes formed by ParB, we next performed analytical size exclusion chromatography coupled to a multi-angle light scattering detector (size exclusion chromatography with multi-angle light scattering (SEC-MALS)) and native mass spectrometry (native MS) (Figure 3). Initial experiments performed in the absence of DNA confirmed that ParB exists as a mixture of monomer and dimer in solution, consistent with previous studies of *T. thermophilus* ParB (Supplementary Figure S3A and B) (15). To investigate the stoichiometry of ParB nucleoprotein complexes, we incubated labelled DNA with approximately equimolar ParB dimers (10-μM DNA :9 μM ParB_2_) and then analysed these samples by SEC-MALS with the detectors set to exclusively monitor the absorbance of the DNA. With 24-bp *pars* DNA (Figure 3A, solid line), the majority of the DNA was found in a single, homogenous peak with a calculated molecular weight of 81.6 ± 1.9 kDa. This value is very close to the expected molecular weight for a ParB dimer bound to a single DNA molecule (79.4 kDa). A lower abundance peak, which eluted earlier than the major peak, was also observed with a calculated molecular weight of 112.1 ± 3 kDa. This peak could represent a trimer of ParB bound to one DNA (109 kDa) or a single dimer bound to three DNAs (111 kDa). In contrast, there was no detectable binding of ParB to a 24-bp non-specific DNA under equivalent conditions (Figure 3A, dotted line). In that experiment, all of the DNA was found in a single late-eluting peak, for which no mass could be assigned, but which we interpreted as free DNA. This was consistent with previous studies of ParB binding to short DNA substrates in gel shift assays in which tight binding was only observed to *parS* DNA (6,12). Non-specific binding of ParB to DNA was however observed by increasing the protein concentration to 80-μM ParB_2_ (Supplementary Figure S3C) and by extending the length of the DNA to either 50 or 100 bp (Supplementary Figure S3D and E; see the Supplementary Information for discussion). Taken together, these data show that ParB binds quite differently to *parS* and to non-specific DNA, especially when the total length of the duplex is relatively short.

The binding of ParB to 100-bp length DNA substrates was also investigated by native MS (Figure 3B). Unlike many other experiments presented here, magnesium was omitted from these experiments as the samples are buffer exchanged into a 300-mM ammonium acetate buffer (pH 7) prior to analysis. Consistent with SEC-MALS, when 5-μM ParB_2_ was incubated with 5-μM 100-bp *parS* DNA, we primarily detected species with molecular weights corresponding to a single ParB dimer bound to DNA (measured 126057 ± 66 Da, calculated 125978 Da) as well as free DNA (61940 ± 68 Da (calculated 61820 Da). Higher molecular weight species were observed (measured ∼195000 Da) but in very low abundance. We also performed experiments with non-specific DNA but were unable to detect binding: only free DNA and protein were visible in the spectra (data not shown).

**Figure 4.**
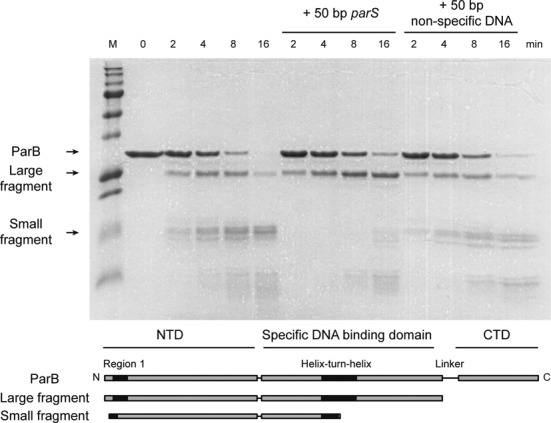
Specific binding of *parS* to ParB protects the helix-turn-helix region from proteolysis. ParB (2-μM dimer) was progressively digested into a large and a small fragment by trypsin, with approximate weights of 26 and 15 kDa, respectively, as determined by a comparison to molecular weight markers. N-terminal sequencing of the excised bands revealed the N-terminal sequences of these fragments to be MAKX and KXIN, respectively. The N-terminus of the large fragment is M1, with the C-terminus lying within the linker region between the central and C-terminal domains of ParB. The N-terminus of the small fragment is K7, which lies within the Box I motif, and the C-terminus is within the helix-turn-helix motif (K132 or K143). The lower panel shows a cartoon representation of the primary structure indicating the major degradation products. In the presence of *parS* DNA (20 μM), the degradation of the large fragment to the small fragment (and therefore cleavage near the helix-turn-helix motif) is substantially reduced, whereas an equivalent non-specific DNA does not have this effect.

To probe for changes to surface accessibility and overall structure upon DNA binding we turned to limited proteolysis (Figure 4). Since solvent exposed cleavage sites are more accessible to trypsin, DNA binding may result in the protection or exposure of trypsin cleavage sites either by steric exclusion or via conformational changes in the protein. ParB (2-μM dimer) was incubated in either the presence or absence of a high concentration (20 μM) of either *parS*-containing or non-specific DNA and then subjected to limited digestion by a low concentration of trypsin. The degradation of ParB was monitored over time by SDS-PAGE, and the molecular weight of the resulting species was estimated using size markers. In the absence of DNA, ParB is progressively degraded into a ladder of smaller species. A semi-stable fragment of ∼26 kDa is formed first, which itself degrades further into a series of smaller fragments including a prominent species of ∼15 kDa. The 26- and 15-kDa fragments will be referred to below as the large and the small fragment, respectively. To identify these fragments we used N-terminal peptide sequencing in combination with their approximate molecular weights. The large fragment was shown to start at the native N-terminus of the protein (Sequencing result: MAKX) and, based on its size, must therefore end in the linker region between the central and C-terminal domains of ParB, which contains several possible cleavage sites. We can conclude that the C-terminal domain is rapidly cleaved from the rest of the protein and degraded under all conditions tested. The N-terminus of the small fragment is K7 (Sequencing result KXIN), meaning that the end of this fragment is either within or very close to the helix-turn-helix motif that contains several possible trypsin cleavage sites (K7 to K132 yields Mw 14.2 kDa or K7 to K143 yields 15.5 kDa).

Importantly, we found that the binding of ParB to *parS* results in a strong stabilization of the large fragment against further degradation, meaning that (minimally) it must prevent cleavage near the helix-turn-helix motifs. This makes good sense, because the helix-turn-helix motif that is found in this region is thought to be responsible for specific binding to *parS*, and would be sterically protected from the activity of trypsin. Interestingly however, the protection afforded by *parS*-containing DNA was not observed in equivalent experiments with non-specific DNA. This is despite the fact that both DNA molecules are present at concentrations well above the observed *Kd* values based on the EMSA and fluorescence assays presented above, conditions under which we would expect non-specific DNA to be occurring. This result implies that the non-specific binding mode of ParB, which appears to be independent of interactions with *parS* thermodynamically (see above and Figure 2), might also be structurally distinct, occurring at a different location on the protein than *parS* binding.

### Non-specific binding of DNA by ParB leads to condensation

**Figure 5.**
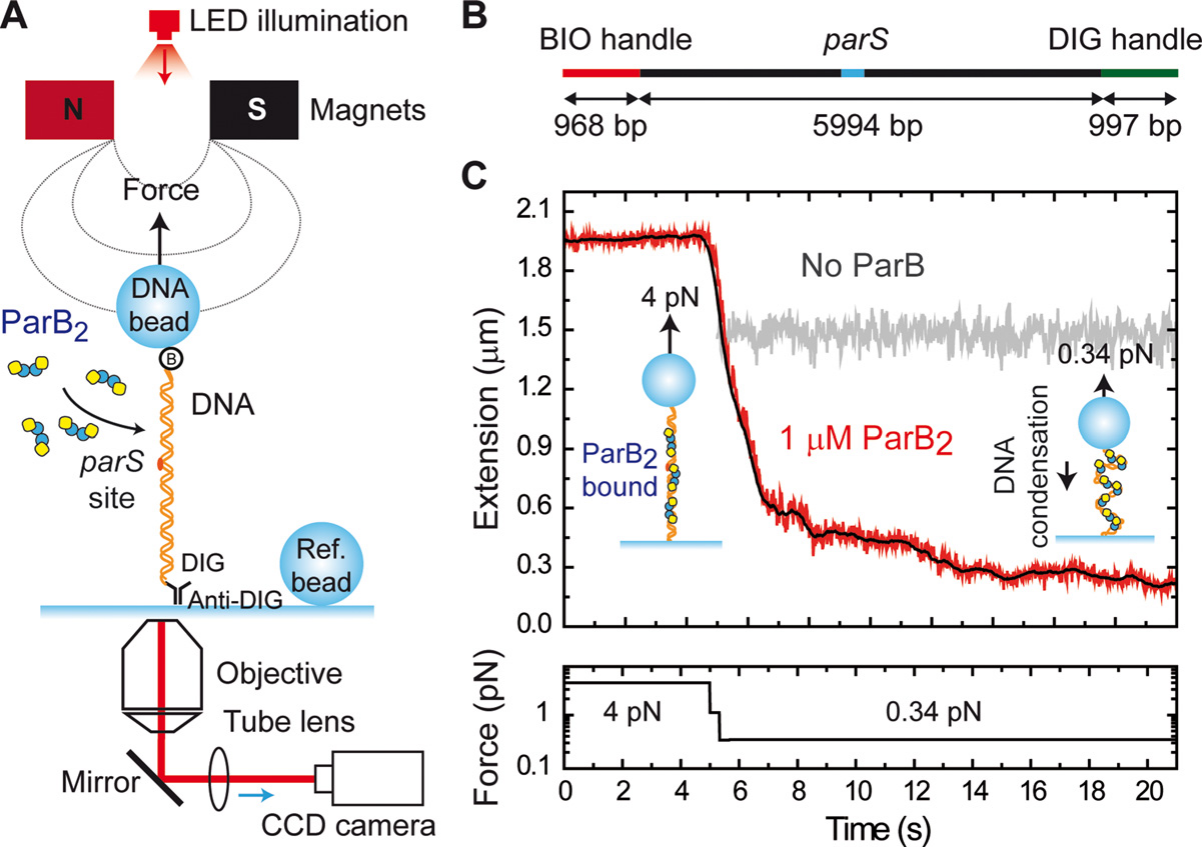
Condensation of DNA by ParB monitored by magnetic tweezers. (**A**) Experimental configuration used to measure condensation dynamics mediated by ParB proteins with magnetic tweezers. (**B**) Schematic representation of the *parS* DNA substrate. (**C**) Condensation assay. At 4-pN stretching force, 1-μM ParB_2_ was injected in the fluid cell and incubated for 2 min. Following incubation, the force was reduced to 0.34 pN. In the absence of protein this leads to the change in extension represented in the grey trace. However, in the presence of ParB we observed a progressive decrease of the extension until reaching a final extension near the surface. Raw data were acquired at 60 Hz (red) and filtered down to 2.4 Hz (black).

Recent studies have shown that ParB is capable of condensing flow-stretched DNA in a single-molecule TIRF apparatus (23). These experiments were carried out under conditions of no free divalent cations, which may prevent specific binding of ParB to *parS* (Supplementary Figure S1). We studied binding of ParB to both *parS*-containing and non-specific DNA in a single-molecule MT setup under conditions suitable for specific binding, and in which the forces associated with condensation and de-condensation can be rigorously investigated (Figure 5A). In these experiments, a DNA substrate of ∼6 kb (Figure 5B) containing a single *parS* site at its centre is attached to a coverslip at one end via DIG:anti-DIG interactions, and to a magnetic bead at the other end by biotin:streptavidin interactions. The bead can then be manipulated by raising, lowering and twisting a pair of magnets above it, and its height is tracked by the diffraction patterns it projects under an LED light. This allows precise control of the force and twist applied to multiple DNA molecules and their heights can be monitored simultaneously. In the absence of ParB, the height of the bead, and therefore the extension of the DNA, is simply dependent on the force applied: a drop in force from 4 to 0.34 pN results in a small drop in extension from ∼1.9 μm to ∼1.5 μm, where the theoretical length of the fully extended substrate is ∼2 μm (Figure 5C). At a constant force of 4 pN, addition of 1-μM ParB_2_ had no effect on the extension of the DNA substrate. However when the force was subsequently dropped to 0.34 pN, we observed a decrease in extension that was very substantially greater than that attributable to the decrease in force alone. The DNA shortened from ∼1.5 to ∼0.2 μm, consistent with the previously reported condensation activity of ParB (23).

**Figure 6.**
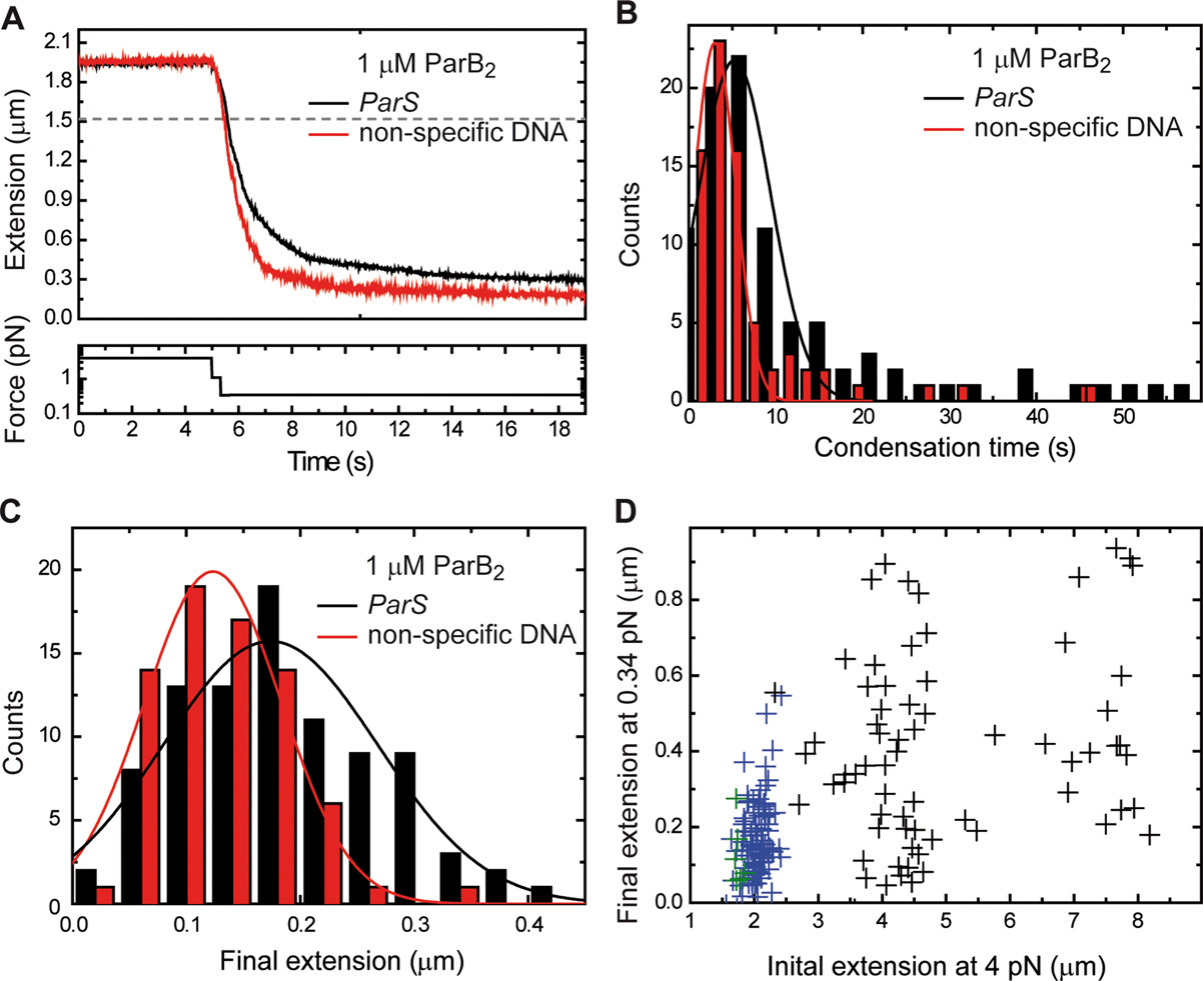
ParB-mediated DNA condensation parameters. (**A**) Mean condensation curves for *parS* (black) and non-specific (red) DNA substrates (*N* > 20). (**B**) Distribution of condensation times for *parS* (black) and non-specific (red) DNA substrates. (**C**) Distribution of final extensions after condensation for *parS* (black) and non-specific (red) DNA substrates. (**D**) Scatter plot of initial and final extensions for lambda-based substrates (black), *parS*-based substrates (blue) and pSP73-based substrates (green). All of the data shown were obtained from condensation curves at 0.34 pN.

Similar results were obtained for a substrate lacking a *parS* site, suggesting that *parS* binding is not required for this activity of ParB (Figure 6A). By combining the observations of multiple beads for the *parS* substrate, we calculated the average time taken for condensation to be 5 ± 4 s, and the average final extension to be 0.18 ± 0.08 μm (Figure 6B and C). We observed a slightly lower condensation time (3±2 s) and final extension (0.12 ± 0.06 μm) for the non-specific DNA substrate, but these differences were within the standard deviation of the data distributions. The final extension and condensation time displayed large variability, with no observable trend at the varying concentrations tested, at least at the force studied (0.34 pN) (Supplementary Figure S4). Importantly, the final extension was not proportional to the length of the DNA substrate used and was highly variable for a given length (Figure 6D). This is inconsistent with a model in which condensation is due to the formation of an ordered species such as a continuous nucleoprotein filament. Freely orbiting MTs experiments that are presented below also support this conclusion.

**Figure 7.**
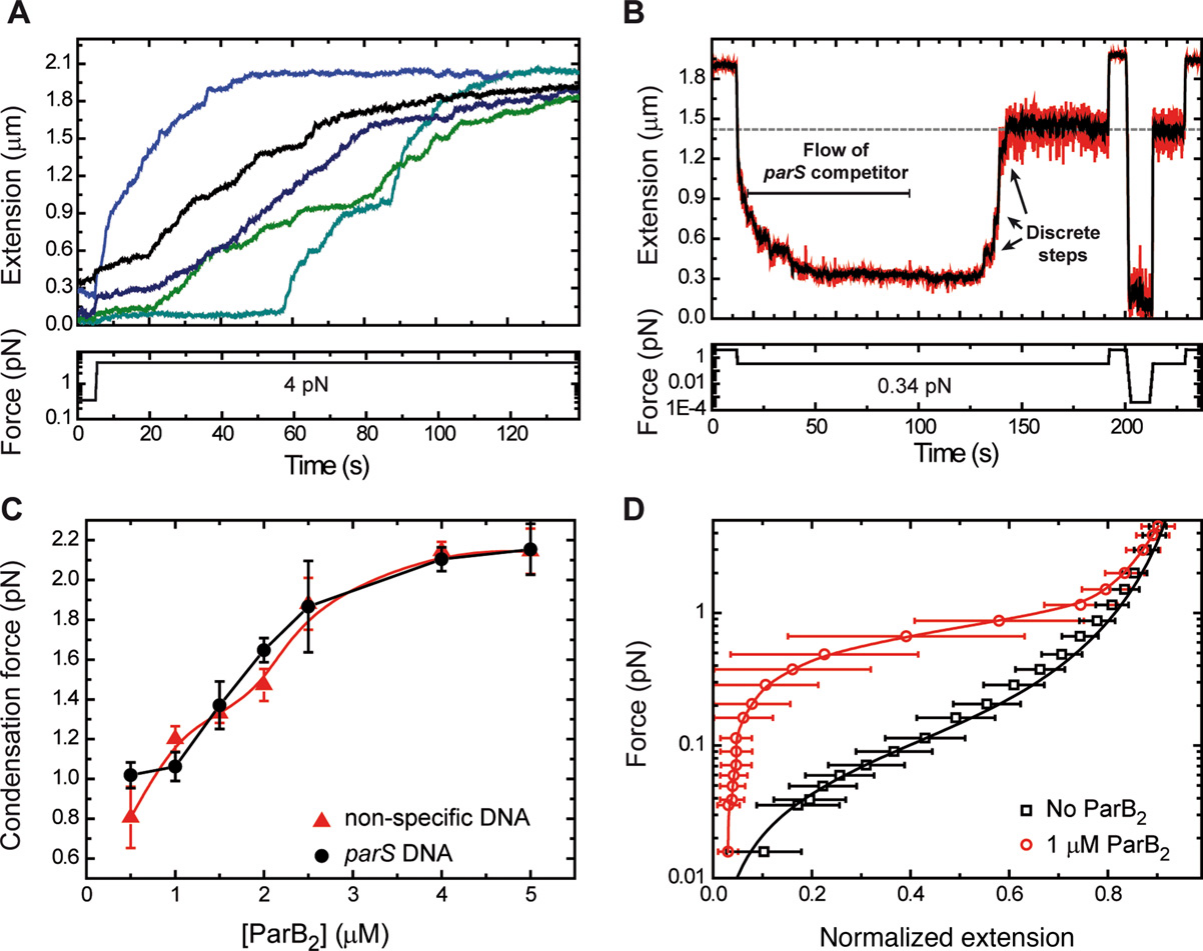
ParB-dependent condensation of DNA is reversible. (**A**) Decondensation of DNA by force. Characteristic force-induced decondensation traces for *parS* substrates are characterized by multiple small steps and a gradual increase of extension. (**B**) Decondensation of DNA by *parS* competitor DNA. Following condensation by reduction of force, a 5-μM *parS* competitor DNA was injected into the flow cell resulting in a process of decondensation characterized by large discrete steps. Decondensation stopped at the extension expected for 0.34 pN applied force in the absence of protein (indicated by the grey dashed line). The lack of protein bound to DNA was checked by raising the force up to 4 pN and reduction down to 0 pN; no (de)condensation effects were observed. (**C**) Condensation force dependency on ParB concentration. A maximum condensation force of 2.1 pN was measured at saturating protein concentration for both *parS* and non-specific DNA substrates. Errors are the standard deviation of measurements on different molecules (*N* ≥ 5 molecules). (**D**) Mean force-extension curve of DNA molecules in the presence of 1-μM ParB_2_ (circles). The solid line is included as a guide for the eye. Data in squares are the control experiment in the absence of protein and the solid line is a fit to the worm-like chain model. Errors are the standard deviation of measurements on different molecules (*N* ≥ 15 molecules).

The condensation process was fully reversible by returning the applied force to 4 pN (Figure 7A and Supplementary Figure S5A), or by the introduction of either *parS* or non-specific competitor oligonucleotides to the flow cell (Figure 7B and Supplementary Figure S5B). However, there are significant differences observed when comparing decondensation curves produced by competitor oligonucleotides versus those produced by application of high force. The former are generally much faster (seconds versus minutes) and are characterized by large steps, whereas the latter are quite variable, lasting from seconds to minutes, and feature many small extension steps. This suggests a substantially different mechanism of de-condensation in each case and this will be discussed below. In either case, the total number of steps required to return to full extension varied substantially between individual experiments, which again suggests that the condensed state is not highly ordered. The presence of competitor DNA in the experiment from the outset prevented DNA condensation indicating that the condensation process was protein-mediated (Supplementary Figures S5C and D).

A single DNA molecule could undergo multiple rounds of condensation and decondensation if the force was repeatedly raised and lowered, with the decondensation traces displaying features that were reproduced in several cycles over the same DNA molecule, but with a degree of variability between different DNA molecules (Supplementary Figures S5E and F). These findings favour a model in which proteins remain non-specifically bound to the DNA at high forces. This phenomenon allowed us to determine the maximum force that prevented condensation at a given [ParB] (see Supplementary Methods for a detailed explanation of how this value is obtained) (Figure 7C). The apparent condensation force exerted by ParB is linear up to concentrations of 2.5-μM ParB_2_, and saturates at ∼5-μM ParB_2_ with a maximum value of ∼2.1 pN. These results are similar to those reported for Fis, another bacterial protein with a role in bacterial chromosome organization (33). They are consistent with the idea that condensation is induced via ParB–ParB interactions, and that these are allowed by DNA fluctuations that are limited by the applied force. For instance, a high force of 2 pN restricts the extension fluctuation of our DNA to 30 nm, and therefore a very high coverage of the DNA (produced by a high concentration of proteins) is required for condensation. The fact that we observe no condensation at forces over 2.1 pN indicates either that the proteins are not close enough to interact, or simply that the protein:DNA and/or protein:protein interactions involved in condensation cannot withstand forces over 2.1 pN.

The process of condensation was further analysed by varying force at a fixed ParB concentration (Figure 7D). The data show that the condensation force at 1-μM ParB_2_ is around 1 pN, and the errors reflect the variability between different molecules employed in this study (*N* = 15). The presence or absence of a single *parS* had no effect on the apparent condensation force induced by ParB (Figure 7C).

### ParB stabilizes looping interactions between distal DNA segments *in cis* and *in trans*

**Figure 8.**
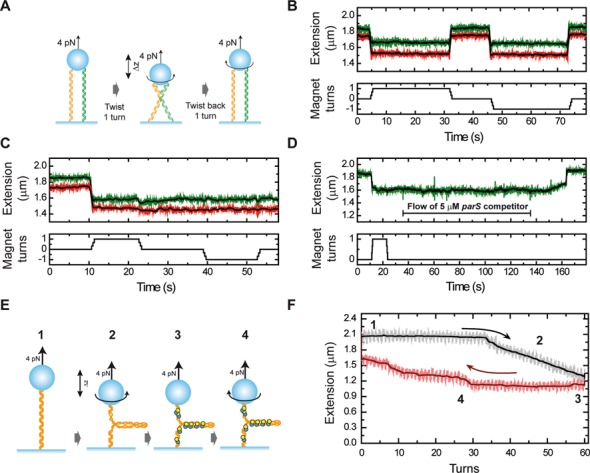
ParB stabilizes crossovers and writhe formed by DNA braiding and bridging. (**A**) Cartoon of the experiment to braid DNA segments *in trans*. The application of one turn (clockwise or anti-clockwise) to doubly tethered beads promotes the cross-over of both DNAs, leading to a change of the extension (Δ*z*). Subsequent untwisting to zero rotation immediately recovers the original extension. (**B**) Time trace of an experiment with two doubly tethered beads recorded simultaneously on bare DNA. (**C**) In the presence of ParB the cross-over is stabilized, and the extension does not recover after untwisting to zero rotation or even when one additional turn in the direction opposite to the cross-over is applied. (**D**) Injection of 5-μM *parS* DNA competitor oligonucleotide promotes the recovery of the full extension following ParB-mediated stabilization of a braid. (**E**) Cartoon of the experiment to bridge DNA segments *in cis*. Single torsionally constrained DNA molecules (1) are positively supercoiled at 4-pN force by applying 60 turns (2). Then, 1-μM ParB_2_ is injected into the fluid cell (3). After full exchange of buffer, all of the turns are released (4). (**F**) DNA extension is displayed as a function of turns to highlight the hysteresis observed due to bridging of different regions of supercoiled DNA after introduction of ParB. The numbers indicate the different stages of the experiment as per the cartoon in part (E).

To test for the formation of ParB-mediated interactions formed between distal DNA segments *in trans*, we next performed a DNA braiding experiment (Figure 8A). In these experiments, a single bead holding two DNA molecules was held at a force non-permissive to condensation (4 pN). Rotation of the magnet in either direction causes the two DNA duplexes to crossover, which is observed as a decrease in the height of the bead. As expected, in the absence of ParB, the removal of this twist unbraids the DNA strands and restores the height of the bead because the two DNA molecules do not interact (Figure 8B). However, in the presence of 1-μM ParB_2_, unbraiding is prevented and the bead does not return to its original height upon untwisting (Figure 8C). This suggests that the two DNA duplexes are held together by ParB. The handedness of the twist had no detectable effect on the stabilization of the braid by ParB (data not shown). The tracking of several of these doubly tethered beads simultaneously showed that this stabilization phenomenon was highly reproducible. The introduction of a *parS*-containing competitor oligonucleotide restored the bead to its original height after untwisting, indicating that braiding-stabilization is dependent on ParB binding to DNA (Figure 8D). Similarly, we found that ParB was capable of stabilizing supercoils introduced by twisting a single torsionally constrained in the magnetic-tweezers setup (Figure 8E and F), suggesting that ParB was bound along the length of the DNA and was stabilizing the plectonemes of the supercoiled DNA by protein–protein interactions *in cis*. Together, these data indicate that ParB is capable of bridging between separate DNA duplexes *in trans*, as well as between different segments of the same DNA molecule.

Finally, to probe the structure of the condensed nucleoprotein complex, we used freely orbiting magnetic tweezers (FOMTs) to provide a measurement of the torque induced by the binding of ParB to torsionally constrained DNA molecules (Supplementary Figure S6A). Prior to any attempt to measure a signal induced by the binding of a protein in the FOMT, we checked that the DNA molecules were torsionally constrained using the standard MT. We also checked that torsionally constrained DNA molecules were condensed by ParB with no detectable differences compared to non-coilable molecules (data not shown). FOMT provided no evidence for concerted twisting of the DNA as condensation occurs (Supplementary Figure S6B), as would be expected for an ordered filament, and as we did observe for the model filament forming recombinase RecA protein using the same apparatus (Supplementary Figure S6C). This result was also confirmed by a topoisomerase-linked bulk assay, in which a closed circular plasmid substrate was incubated with eukaryotic topoisomerase I and increasing concentrations of ParB (Supplementary Figure S6D). If ParB was inducing twist or wrapping the DNA then this would have been evidenced by the insertion of supercoils by topoisomerase I, as seen, for example, in similar experiments on nucleosome binding (34). No change in the supercoiling state was observed with up to 1-μM ParB_2_, suggesting that its binding was not perturbing the superhelical density of plasmids.

## DISCUSSION

ParB forms large nucleoprotein complexes that spread for several kilobase pairs around individual *parS* sites *in vivo*. These act as markers for active segregation and as loading sites for SMC complexes. The mechanisms for ParB spreading, recruitment of SMC complexes and for the ensuing segregation and condensation of the bacterial chromosome are not fully understood. In this work, we studied the binding of purified ParB protein to DNA *in vitro*. EMSAs revealed a clear specificity for binding to the *parS* sequence, because a well-resolved shifted complex in the middle of the gel was only ever observed for *parS*-containing DNA. SEC-MALS and native mass spectrometry demonstrated that the complex formed contained two ParB polypeptides bound to a single *parS*-containing DNA molecule. This complex is likely to represent a C_2_ rotationally symmetric dimer of ParB with an interface in the central region, allowing the binding of the palindromic *parS* sequence across the helix-turn-helix motifs (15). Interestingly, we found no evidence for specific binding to *parS* sequences when employing native gels that were run in an EDTA-containing buffer, as in many previous studies of ParB (6,10,12,23). Therefore, experiments investigating *parS* binding that have been conducted in this manner should be interpreted with caution. At elevated ParB concentrations we also observed a qualitatively different binding of DNA, to form nucleoprotein complexes that are almost unable to migrate into native gels. This phenomenon appeared to be entirely non-specific, as it occurred equally effectively with either *parS*-containing or *parS*-free substrates. For reasons that are developed further below, we speculate that this binding might occur at a second and independent DNA binding locus in ParB. In that scenario, it should be noted that whilst the *overall* specificity for binding to *parS* we observe is low (∼2–3-fold), the secondary binding event that results in the formation of large complexes might mask a potentially high binding specificity at the helix-turn-helix motifs.

Non-specific binding also gives rise to a significant fluorescence signal from end-labelled DNA substrates. This more quantitative binding assay suggested that the non-specific binding is strongly co-operative (Hill co-efficient ∼3–4) on the substrates used here, consistent with their apparently large size. An alternative explanation for the apparent co-operativity is simply that binding to the DNA end is disfavoured compared to internal sites, but the transition to the high fluorescence state matches well with the supershift to larger complexes in gels arguing that this is not the case. Importantly, there is no substantial effect of *parS*-bound ParB in recruiting additional ParB molecules to bind to DNA non-specifically: the binding isotherms obtained for specific and non-specific DNA substrates are the same within the error of the measurement. These findings were additionally corroborated in single molecule MT experiments by showing similar condensation curves for *parS* and non-specific DNA substrates. Interestingly then, it has so far not been possible to re-capitulate the ‘spreading’ phenomenon that has been observed *in vivo* using *in vitro* techniques. This might either reflect the design of the experiment (for example, it is difficult to mimic the chromosomal DNA substrate and its concentration *in vitro*) or that we are missing additional components that mediate the spreading.

We found that the C-terminal region of ParB was highly susceptible to removal by proteolysis, consistent with existing structural models (15). Additionally, the remaining central region of the protein was strongly protected from further proteolysis by the specific binding of *parS*. This is consistent with the idea that the helix-turn-helix motifs found in this core part of the protein are important for binding *parS*, as predicted in previous work (15). Remarkably, binding of ParB to a non-specific DNA substrate afforded no such protection at the highest concentration tested. This might suggest that non-specific DNA binds at a different position on the ParB protein. There is currently no direct evidence for a second DNA binding locus for non-specific DNA in *B. subtilis* ParB. However, it is clear from studies of some plasmid-encoded ParB proteins that formation of the partition complex involves multi-modal interactions with DNA at different binding loci, which in part reflects the variety and relative complexity in the architecture of the *parS* sites that they recognize. For example, in the P1 ParB system, the interface formed by the C-terminal dimerization domain binds to two ‘B-box’ sequences via DNA binding ‘wings’ (19). However, in the F plasmid SopB protein these DNA binding wings are not present in the C-terminal domains, although a non-specific DNA binding site was identified (20). It will be of significant interest to determine whether *B. subtilis* ParB harbours a non-specific DNA binding site that is structurally distinct from the helix-turn-helix motif.

Recent work using single molecule visualization and complementary modelling of ParB binding to DNA has shown that ParB is capable of condensing DNA, and suggests that this activity is the result of bridging interactions between many non-specifically bound ParB molecules (23,24). Consistent with these data, we found using MTs that ParB was able to condense DNA against a maximum applied force of 2.1 pN. This is a fairly small force that could easily be applied by the many cellular motor proteins that act on DNA. An attractive idea, therefore, is that ParB-mediated condensation may be modulated by other processes that remodel bacterial chromatin via stretching of the chromosome. Condensation was dependent on the ParB–DNA interaction as it was both inhibited by and fully reversible through the addition of free DNA. Condensation was also reversible by applying a large force, although these decondensation traces were markedly different from those obtained by the addition of competitor DNA. The different behaviour in each case is consistent with a model in which multiple ParB protomers stabilize large loops of DNA in the condensed state. In that case, decondensation by force would selectively disrupt the ParB–DNA interactions at the edges of such structures, leading to DNA extension in small steps as the loops become progressively smaller. In contrast, addition of competitor DNA would randomly disrupt the interactions, such that when the critical interactions at the edge of condensed loops are eventually eliminated, they are likely to release larger amounts of DNA, leading to extension in bigger steps as observed (see Supplementary Figure S7 for a cartoon). A model in which ParB bridges distant DNA segments to form loops is further supported and extended by our observations that ParB can stabilize writhe in supercoiled DNA molecules, and can hold together braids between two DNA molecules. Importantly, these experiments also exclude the possibility that the condensation activity we observe is due to interactions of ParB with the surface of the flow cells used in our apparatus. Additionally, we found that the condensed DNA structure formed by ParB was not well ordered. The final extension was highly variable and only weakly correlated with the initial DNA length. Moreover, freely orbiting MTs experiments showed that condensation is not coupled to DNA twisting in a concerted fashion, as is the case for classical nucleoprotein filament forming proteins such as RecA. We conclude that *Bacillus* ParB (a Type Ia centromere binding protein) probably forms structures that are less ordered than Type Ib complexes (31,35), and the large ‘superhelix’ complexes that have been suggested for some Type II systems such as pSK41 ParR (14,36).

**Figure 9.**
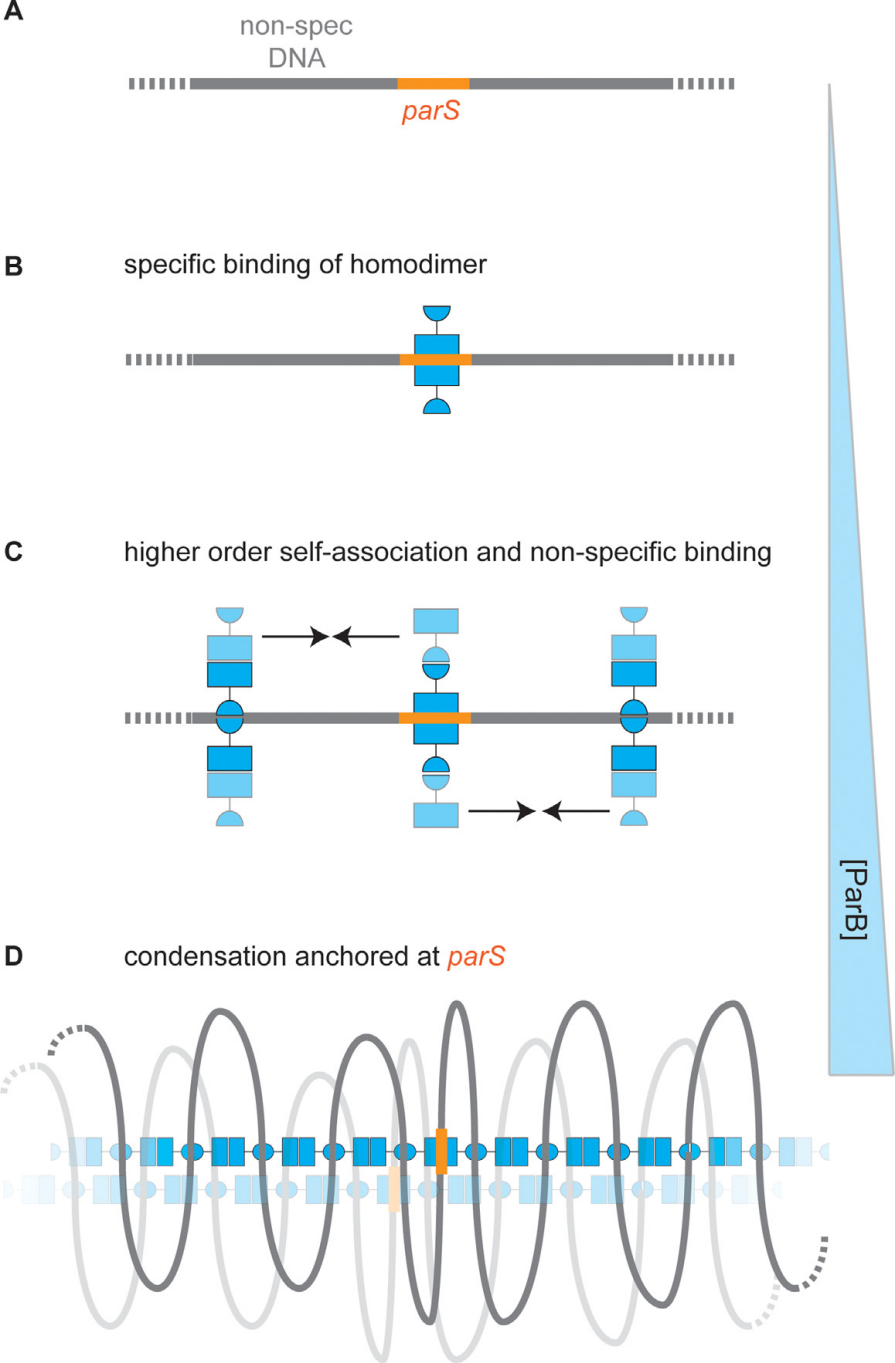
Specific and non-specific binding of DNA by ParB: a speculative model for spreading at *parS* sites. (**A**) A region of a DNA molecule containing a specific binding site is shown. (**B**) Specific binding. At low concentrations, ParB binds to *parS* sequences via the central helix-turn-helix motifs to form a ParB_2_:DNA complex (supporting data in Figures 1, 3 and 4). (**C**) Non-specific DNA binding. Elevated concentrations of ParB allow co-operative non-specific binding via a second (hypothetical) DNA binding domain (supporting data in Figures 1 and 2). The continued self-association of ParB (indicated with arrows) via at least two interfaces subsequently leads to formation of higher order networks and DNA condensation. This transition is not dependent on the presence of *parS*. (**D**) The condensed nucleoprotein network (supporting data in Figures 5–8) may contain both specific and non-specific DNA binding sites (see main text for justification) that trap loops of DNA that are anchored around *parS* if the *parS* site is present. For simplicity, the specific binding sites for most of the ParB dimers are shown unoccupied. Such structures might bridge larger distances, including between distant *parS* loci, through the sharing of segments of DNA, or via additional protein:protein interactions (indicated with the faded nucleoprotein complex).

A working model for the binding of ParB at and around *parS*, leading to localized condensation of DNA, is shown in Figure 9. It is somewhat similar to recently published models (23,24), which explained how large regions of DNA around *parS* could be condensed by relatively few ParB protomers and rationalized the effects of roadblocks on ParB distribution in terms of a bridging model (as opposed to the, perhaps more intuitive, lateral spreading or filament model). That work envisioned that the ParB protein contained a single DNA binding site, and therefore assumed that the non-specific DNA binding required for looping and spreading must be competitive with specific binding. In this single DNA binding site scenario, given the very high concentration of non-specific versus specific sites *in vivo*, localization of the ParB nucleoprotein complexes at *parS* would require an exceptionally high degree of specificity of binding to *parS* sequences. However, this is not well supported by the available data for the behaviour of ParB *in vitro* which shows that non-specific binding occurs at fairly low concentrations (6,12). In fact, the work presented here shows no apparent difference between specific and non-specific binding affinity at all under conditions of no free divalent cations, and less than a 10-fold difference under more physiological conditions. Whilst this apparent lack of high specificity might simply reflect the limitations of *in vitro* assays, it is also important to recognize that, under the latter conditions, the non-specific binding is qualitatively different from, and independent of, the *parS* binding. Therefore, an alternative idea is that the non-specific binding we observe may occur at a dedicated and structurally distinct DNA binding locus, as is the case for other Type 1a centromere binding proteins (14). Such a binding site might belie a potentially very high specificity for binding *parS* at the helix-turn-helix motifs. This idea is supported by the lack of protection from proteolysis that is afforded to these motifs by non-specific DNA binding, which is in contrast to the case with *parS* binding. In such a scenario, large protein networks formed by ParB protein through its self-association interfaces would contain both highly specific *parS* binding sites and additional non-specific sites (Figure 9C and D). DNA condensation might require the co-operative binding of multiple ParB proteins present at relatively high concentration and could occur anywhere on the chromosome. However, in a situation where the total protein is limited relative to DNA binding sites, as is the case *in vivo*, these networks would be preferentially anchored around *parS* by the additional binding energy available at the specific binding locus. These ideas will be tested and developed further in future work.

## SUPPLEMENTARY DATA

Supplementary Data are available at NAR Online.

SUPPLEMENTARY DATA
