# Supplementary material for: Specific and non-specific interactions of ParB with DNA: implications for chromosome segregation
Source: Nucleic Acids Res. 2015 Jan 8;43(2):719–31. doi: 10.1093/nar/gku1295 (PMC4333373; doi:10.1093/nar/gku1295)
Supplement: SUPPLEMENTARY DATA [file supp_43_2_719__index.html]

Specific and non-specific interactions of ParB with DNA: implications for chromosome segregation — Specific and non-specific interactions of ParB with DNA: implications for chromosome segregation — SUPPLEMENTARY DATA 

# Specific and non-specific interactions of ParB with DNA: implications for chromosome segregation

## SUPPLEMENTARY DATA

**Files in this Data Supplement:**

- SUPPLEMENTARY DATA
