# Supplementary material for: Specific and non-specific interactions of ParB with DNA: implications for chromosome segregation
Source: Nucleic Acids Res. 2015 Jan 8;43(2):719–31. doi: 10.1093/nar/gku1295 (PMC4333373; doi:10.1093/nar/gku1295)

Specific and non-specific interactions of ParB with DNA: implications for chromosome segregation

James A. Taylor, Cesar L. Pastrana, Annika Butterer, Christian Pernstich, Emma Gwynn, Frank Sobott, Fernando Moreno-Herrero and Mark S. Dillingham

Supplementary Information

Supplementary Methods

Supplementary References

Supplementary Table 1

Supplementary Figures Legends

Supplementary Figures S1-S7

Supplementary Methods

ParB-DNA association kinetics

For assays monitoring the kinetics of ParB binding to Cy3-labelled DNA via changes in fluorescence, ParB (at double the concentration indicated) was prepared in a buffer containing 50 mM Hepes·KOH (pH 7.5), 100 mM KCl, 2.5 mM $MgCl_2$, 0.1 mg/mL BSA and 1 mM DTT. A 50 µl aliquot of this sample was then rapidly mixed in a stopped-flow apparatus (TgK Scientific) with an equal volume of 40 nM 147 bp Cy3-labelled DNA substrate (see **Supplementary Table 1**), which was prepared in a buffer identical to the one described above. This yielded a final solution with 20 nM 147 bp Cy3-labelled DNA and the indicated concentration of ParB. The sample was excited at 549 nm and the emission recorded with a bandpass filter of >570 nm over time. Three repeats were performed for each concentration of ParB and the mean values plotted.

Plasmid topology assay

To determine if binding of ParB to DNA had a significant effect on DNA twist or writhe a topoisomerase I–linked assay was performed. The construct of plasmid pSP73 containing a *parS* site was relaxed by the addition of wheat germ Topoisomerase I (Inspiralis Ltd). Relaxed plasmid was then incubated for a further 30 min at 37 °C with 2 U wheat germ topo I and a titration of ParB. The reaction was stopped by the addition of 0.1% SDS and the proteins removed by phenol:chloroform extraction before the samples were run on a 1% agarose gel at 80 V in TBE buffer for 3 hrs. The DNA was then visualised by ethidium bromide staining, and the samples inspected for a ParB-dependent change in plasmid topology.

Calculation of the duration and extent of condensation

The final extension (Z_{end}) (**Figure 6C**) was the mean extension in a time window of two seconds at the end of a condensation event. We considered that a condensation event finished when there are no visible changes in extension for several tens of seconds. The condensation time (**Figure 6B**) was determined as the time to reach an extension given by Z_{end} - 2 σ_{end}, where σ_{end} is the standard deviation of the extension at the end of the condensation process. This criterion eliminated small condensation events occurring after long periods of time that would otherwise skew the time distribution to longer values.

Condensation force

To determine the force at which the process of condensation starts (**Figure 7C**), the magnet was alternated between positions that apply a large stretching force (3.9 pN) or a variable smaller force that was gradually decreased. The low force was maintained for 35 s and the mean extension for the first (E_0) and the last five seconds (E_f) of the measurement determined. The condensation force is defined as the critical force that satisfies the following criterion:

$$E_f < E_0 - 10\sigma_0$$

where σ_0 is the standard deviation of E_0 at 1 Hz bandwidth. This threshold value ($10\sigma_0$) allowed us to discriminate between real condensation events and oscillations that are sometimes visible at forces near condensation.

Force-extension curves

For each DNA molecule, a value of the extension at a given force was calculated (**Figure 7D**). Then, a mean value and standard deviation were determined for multiple DNA molecules. In the absence of protein, data were fitted to the worm-like chain model (1). From the fits, we obtained the contour length (L_0) of the molecule, which was subsequently used to normalize the data, and the persistence length (P). We measured a mean L_0 and P of 2.2 µm and 33 nm. The value of P was smaller than previously reported (2). This could be due to the presence of Mg^{2+} in the buffer (3) and/or to the variability in the attachment positions of the DNA to the magnetic bead in parallel measurements. The molecule previously characterized in the absence of protein was then studied in the presence of 1 µM $ParB_2$. ParB force extension data was calculated as above but was not fitted to any model. The solid red line in **Figure 7D** is simply included as a guide for the eye.

Freely-Orbiting MT (FOMT) experiments

We used a magnet arrangement similar to that described in (4). In this setup, superparamagnetic beads are free to rotate in a plane perpendicular to the DNA axis, thus providing a measurement of the torque induced by the binding of proteins to torsionally constrained DNA molecules. Thus, prior to any attempt to measure a signal induced by the binding of a protein in FOMT, we checked that the DNA molecules were torsionally constrained using the standard MT described in the Materials and Methods section. As in the standard MT, this setup also provides the extension of the DNA in real time. As a control experiment, we reproduced the assay reported in (4) with RecA protein using the buffer 20 mM Hepes pH 6.0, 50 mM NaCl, 1 mM $MgCl_2$, 0.1 mM ATPgS, 1 mM DTT, 100 µg/ml BSA, 0.1% TWEEN 20.

Supplementary References

1. Bouchiat, C., Wang, M.D., Allemand, J., Strick, T., Block, S.M. and Croquette, V. (1999) Estimating the persistence length of a worm-like chain molecule from force-extension measurements. *Biophysical journal*, **76**, 409-413.
2. Bustamante, C., Marko, J.F., Siggia, E.D. and Smith, S. (1994) Entropic elasticity of lambda-phage DNA. *Science*, **265**, 1599-1600.
3. Baumann, C.G., Smith, S.B., Bloomfield, V.A. and Bustamante, C. (1997) Ionic effects on the elasticity of single DNA molecules. *Proceedings of the National Academy of Sciences of the United States of America*, **94**, 6185-6190.
4. Lipfert, J., Wiggin, M., Kerssemakers, J.W., Pedaci, F. and Dekker, N.H. (2011) Freely orbiting magnetic tweezers to directly monitor changes in the twist of nucleic acids. *Nature communications*, **2**, 439.

Supplementary Table 1: Oligonucleotides used in this study

**Use**	**Direction**	**Modification (if present)**	**Sequence (5'-3')**
ParB cloning primer	Forward		GTG AGA CCA TGG CTA AAG GCC TTG GAA AAG
ParB cloning primer	Reverse		CAA TTC GGA TTC TTA TGA TTC TCG TTC AGA CAA AAG
Removal of NotI and XhoI sites from pET28A(+)	Forward		GTC GAC AAG CTT CCG GCCGCA GTC GAG CAC
Removal of NotI and XhoI sites from pET28A(+)	Reverse		TGC TCG ACT GCG GCC GGA AGC TTG TCG ACG
147 bp substrate primer	Forward	5'-Cy3	GGT ACA AAA AGT GAT TGC G
147 bp substrate primer	Reverse		TTT TGG AGA TAG GAT TCC C
24 bp parS	Forward	5'-Hex	AGA A**TG TTC CAC GTG AAA CA**A AGA
24 bp parS	Reverse		TCT T**TG TTT CAC GTG GAA CA**T TCT
24 bp non-specific	Forward	5'-Hex	AGA ACG TGC CCA GGG AGA CAA AGA
24 bp non-specific	Reverse		TCT TGC ACG GGT CCC TCT GTT TCT
50 bp parS	Forward	5'-Hex	CAG CAG TTG AAT CAG AAT **GTT CCA CGT GAA ACA** AAG AAA AAA GAA CCT GT
50 bp parS	Reverse		ACA GGT TCT TTT TTC TT**T GTT TCA CGT GGA ACA** TTC TGA TTC AAC TGC TG
50 bp non-specific	Forward	5'-Hex	CAG CAG TTG AAT CAG AAC GTG CCC AGG GAG ACA AAG AAA AAA GAA CCT GT
50 bp non-specific	Reverse		TCT TTG TTT CAC GTG GAA CAT TCT ACA GGT TCT TTT TGA TTC AAC TGC TG
100 bp parS	Forward	5'-Hex	TGT TCG CCA ACT TGA GCA GCT GAT TCA GCA GTT GAA TCA GAA **TGT TCC ACG TGA AAC A**AA GAA AAA AGA ACC TGT GAA AGA TGC

			GGT TCT AAA AGA ACG G
100 bp parS	Reverse		CCG TTC TTT TAG AAC CGC ATC TTT CAC AGG TTC TTT TTT CTT **TGT TTC ACG TGG AAC A**TT CTG ATT CAA CTG CTG AAT CAG CTG CTC AAG TTG GCG AAC A
100 bp non-specific	Forward	5'-Hex	TGT TCG CCA ACT TGA GCA GCT GAT TCA GCA GTT GAA TCA GAA CGT GCC CAG GGA GAC AAA GAA AAA AGA ACC TGT GAA AGA TGC GGT TCT AAA AGA ACG G
100 bp non-specific	Reverse		CCG TTC TTT TAG AAC CGC ATC TTT CAC AGG TTC TTT TTT CTT TGT CTC CCT GGG CAC GTT CTG ATT CAA CTG CTG AAT CAG CTG CTC AAG TTG GCG AAC A
MT substrate fabrication (handles)	Forward		GCGTAAGTGCGGCCGCGACTCACTATAGGGAG ACCGGC
MT substrate fabrication (handles)	Reverse		AGTAAGCGCCGTCAGACCAG
FMH-F-NotI-pET28-SpoOJ<br><br>MT substrate fabrication (central region)	Forward		GCGTAAGTGCGGCCGCCTGCATGTGTCAGAGG TTTTCAC
FMH-R-XhoI-pET28-SpoOJ<br><br>MT substrate fabrication (central region)	Reverse		GCGTAAGTCTCGAGCAGATTGTACTGAGAGTGC ACCA

Supplementary Figure Legends

Figure S1. Specific and non-specific binding of ParB to DNA monitored by EMSA: effect of gel buffer composition. (A) ParB dimers (at the concentration indicated) were incubated with a 147 bp DNA molecule (20 nM) that either did (left), or did not (right), contain a central *parS* sequence in standard reaction buffer. The protein:DNA complexes were then resolved on EDTA-containing (TBE gel) native polyacrylamide gels. This protocol is similar to those used in previous reports (see main text for discussion). Under these conditions and in our hands, there is no qualitative or quantitative difference between the ParB-DNA complexes formed on the two different DNA molecules. In both cases, there is a ladder of shifted complexes, displaying apparently larger molecular weights at higher [ParB]. This data is broadly consistent with non-specific and non-cooperative coating of the available binding sites on the DNA molecule regardless of the presence of *parS*, and consequently there is no evidence for specific binding in this experiment. **(B)** The experiment shown in part A was repeated, but the complexes were resolved in a gel containing free magnesium ions (TBM gel). These experiments are equivalent to those shown in **Figure 1** of the main manuscript, and reveal a specific interaction with the *parS*-containing DNA leading to the formation of a well-resolved band in the middle of the gel. At elevated [ParB], both DNA molecules are bound to form non-specific complexes that are poorly resolved by the gel.

Figure S2. Association kinetics for the formation of non-specific ParB-DNA complexes. ParB protein (at the final concentration indicated) was rapidly mixed with a 147 bp DNA molecule that either did **(A)** or did not **(B)** contain a single central *parS* sequence. The molecule was also end-labelled with a Cy3 fluorophore which reports non-specific binding of ParB, presumably when it is close to the label (see main **Figure 2** and main text for discussion). As expected based on the binding isotherms presented in the main paper, low concentrations of ParB result in a barely detectable binding signal. At higher concentrations of ParB, the formation of non-specific complexes (as monitored by the evolution of the fluorescence increase) is slow and complex. Importantly, there is no substantial difference in the kinetics if the *parS* sequence is present or not.

Figure S3. SEC-MALS and native MS analysis of ParB and ParB-DNA complexes with different DNA lengths. (A) ParB protein alone analysed by SEC-MALS produces a single peak, with a calculated molecular mass approximately consistent with a dimeric species. **(B)** Native MS analysis of ParB alone detects monomeric and dimeric species in approximately equal quantities. **(C-E)** Analysis of complexes formed by incubating ParB (80 µM) with DNA (10 µM, the DNA length is indicated). In each case the DNA either does (black line) or does not (red line) contain a single central *parS* sequence. In all cases, binding of ParB to DNA is detected. For longer DNA molecules (50 and 100 bp) the traces are similar regardless of the presence or absence of *parS*, and the molecular weights of the complexes formed are large but poorly defined. Note that, for the shortest duplex tested (24 bp), the complexes formed are distinct (see main text for further discussion).

Figure S4: Characterisation of ParB-dependent condensation of *parS* and non-specific DNA substrates

(A) Representative condensation curves for a *parS*-containing DNA substrate. **(B)** Representative condensation curves for a non-specific DNA substrate. The dashed line represents the expected extension for a force of 0.34 pN in the absence of protein. The presence of *parS* makes no apparent

difference to the process of ParB-dependent condensation of DNA. **(C)** Condensation times measured for different protein concentrations. The data were obtained using the *parS* DNA substrate. **(D)** Final extension measured for different protein concentrations. The data were obtained using the *parS* DNA substrate. In panels C and D, each black line represents an individual measurement from separate condensation curves and the red line is the average value for all molecules.

Figure S5: ParB-dependent condensation of DNA is reversible, and inhibited, by addition of free DNA molecules

(A) Characteristic force-induced decondensation traces for non-specific DNA substrates **(B)** Decondensation of *parS* DNA substrate by non-specific competitor DNA. Following condensation by reduction of force, a non-specific competitor DNA (50 bp, 5 μM) was injected into the flow cell resulting in a process of decondensation characterized by several big steps and a sudden increase of extension. Injection of *parS* DNA typically resulted in a faster decondensation process compared to non-specific DNA (see main text **Figure 7B**). The expected extension for 0.34 pN in the absence of protein is shown by the grey dashed line and is reached after decondensation by the DNA competitor. The absence of protein remaining bound to the DNA was further checked by raising the force to 4 pN and subsequent reduction down to 0 pN: no (de)condensation effects were observed. **(C)** Condensation was inhibited by the presence of *parS* competitor DNA from the outset of the experiment. **(D)** Condensation was also inhibited by the presence of non-specific competitor DNA, although a larger amount of was required to prevent condensation in comparison with specific *parS* competitor. **(E)** Force-stretching cycles showing the reversibility of ParB-dependent condensation of *parS* DNA. **(F)** Force-stretching cycles showing the reversibility of ParB-dependent condensation of *non-specific* DNA.

Figure S6: Freely-orbiting magnetic tweezers and bulk topology experiments show no evidence for the formation of an ordered ParB nucleofilament

(A) Experimental configuration in freely orbiting magnetic tweezers, where the use of a toroidal magnet allows the bead to rotate in a plane perpendicular to the DNA axis. **(B)** ParB-dependent condensation assay using freely orbiting magnetic tweezers and torsionally-constrained DNA molecules. The turns made by the bead during condensation are both clockwise and anti-clockwise, small in number (<6 at most), and uncorrelated with changes to extension. **(C)** This behavior of ParB is in stark contrast to that displayed by an ordered filament-forming protein such as RecA, where directional untwisting is tightly correlated with DNA extension, as expected based on previous work (ref). **(D)** A DNA topology assay shows no evidence for ParB-dependent changes to superhelical density in a closed circular DNA molecule (see **Supplementary Methods** for details, and main text for discussion).

Figure S7: Model to explained observed differences between DNA decondensation by force compared to competitor DNA

(A) ParB proteins bind non-specifically to a DNA molecule held at 4 pN force. **(B)** ParB-dependent condensation is triggered at low force. Condensation is reversible upon application of force (route i) or by addition of a competitor DNA (route ii). **(C)** Decondensation by force preferentially disrupts ParB-DNA interactions at the edges of the loops, resulting in DNA extension in many small steps. **(D)**

Decondensation by addition of competitor DNA *randomly* disrupts ParB-DNA interactions such that **(E)** decondensation only occurs when a critical interaction at the edge of a condensed loop (purple circle) is released by chance, and a much bigger step is likely to be observed.

Taylor et al. Figure S1

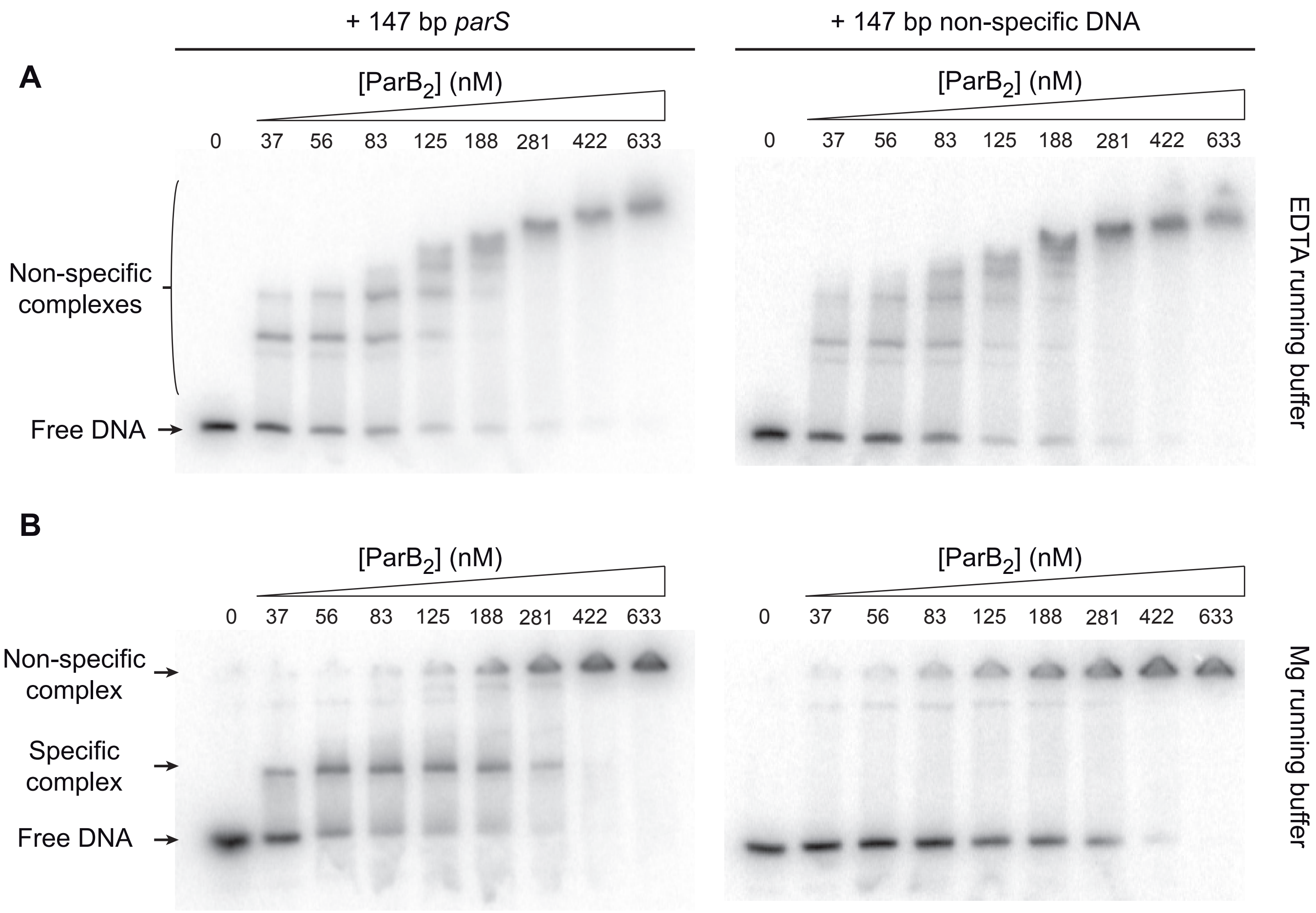

Taylor et al. Figure S2

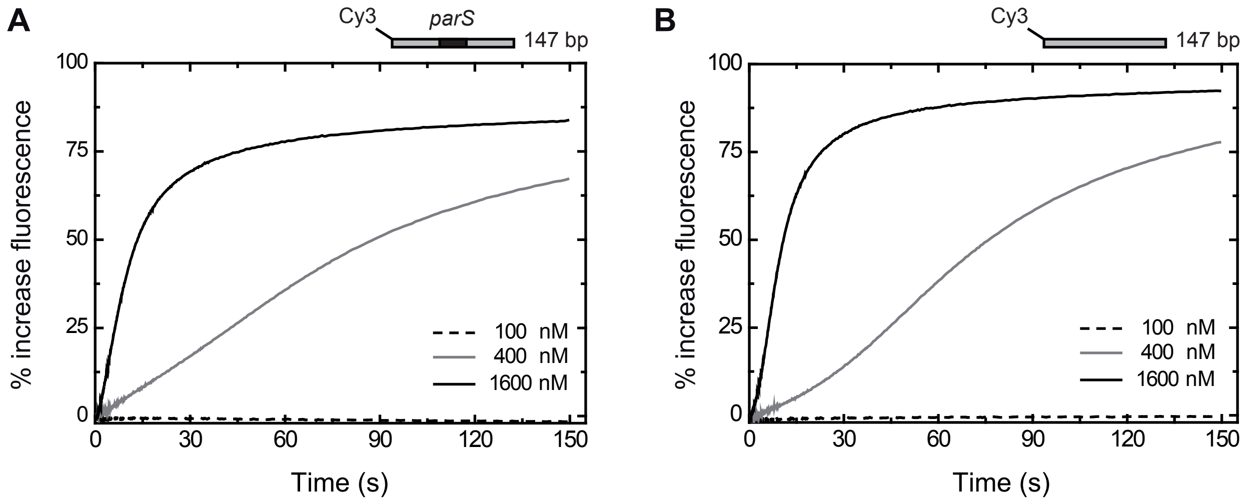

Taylor et al. Figure S3

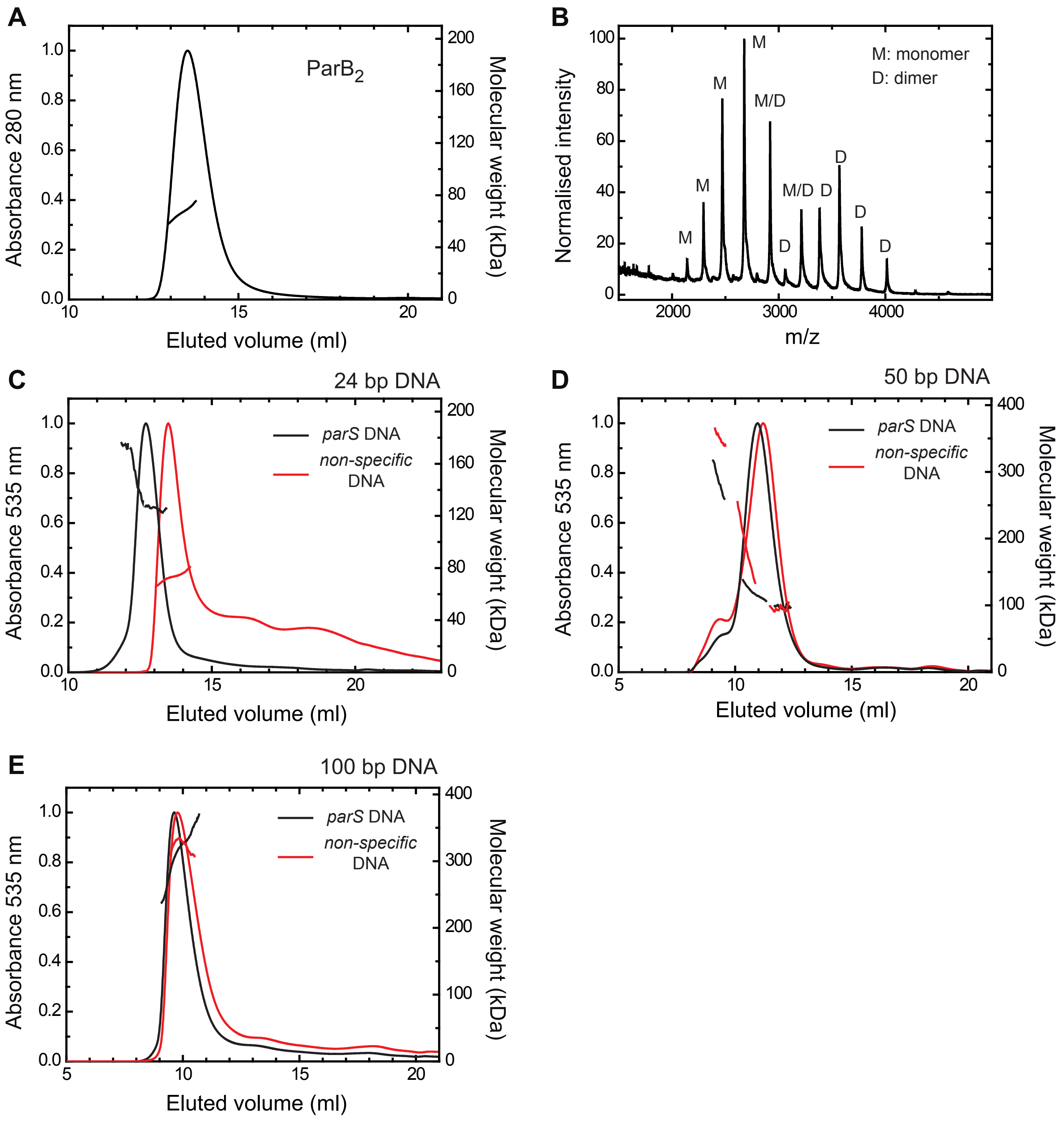

Taylor et al. Figure S4

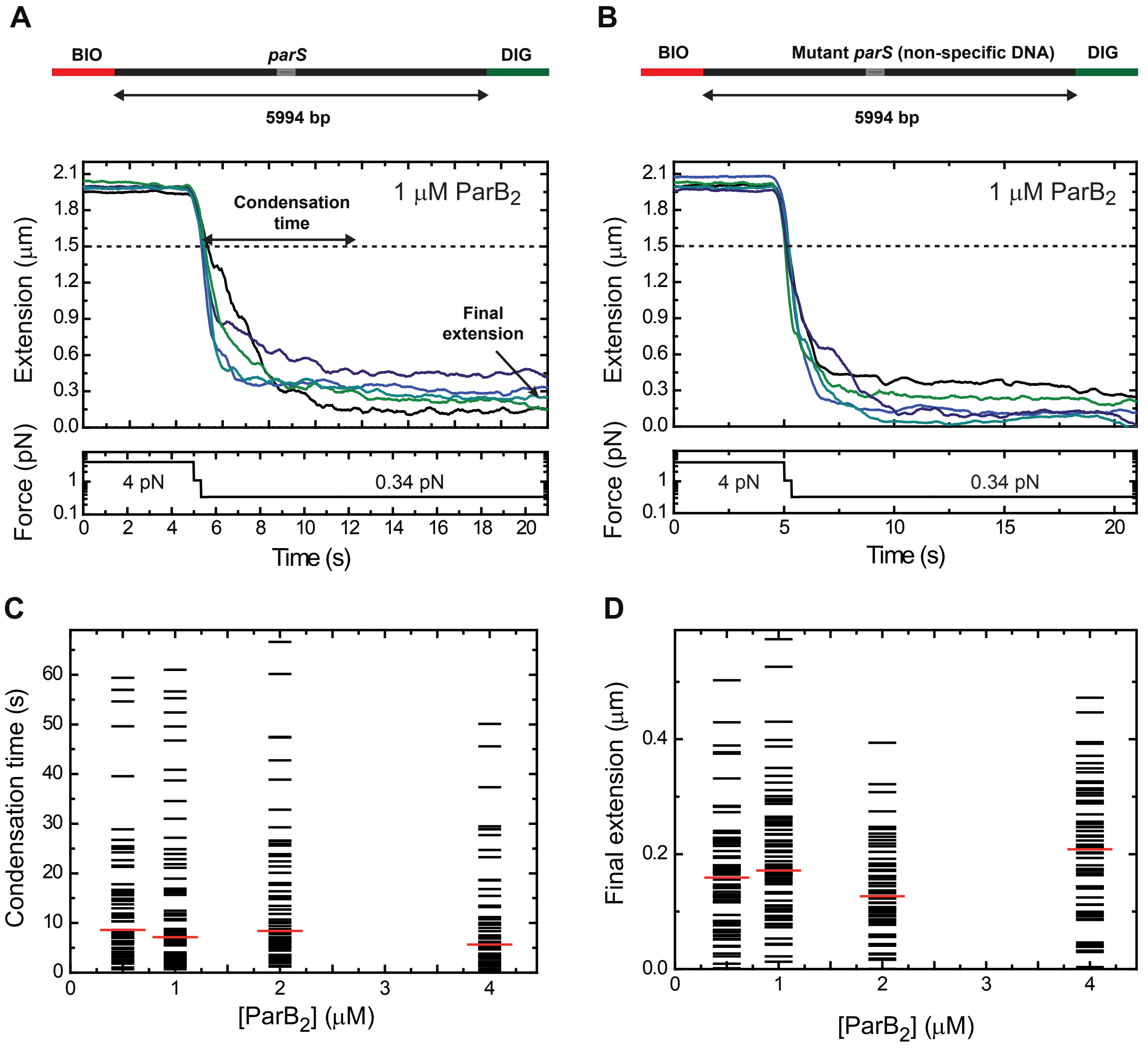

Taylor et al. Figure S5

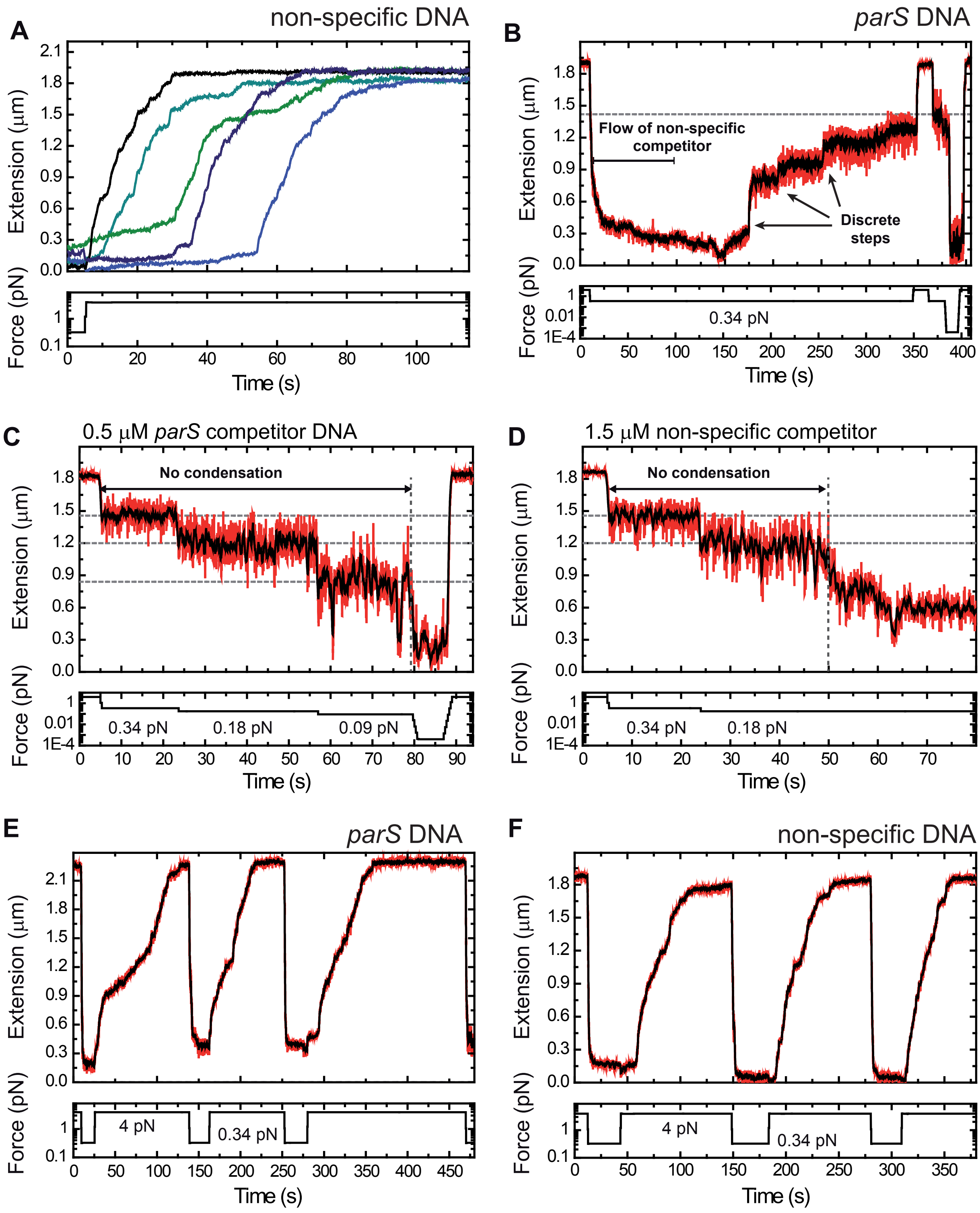

Taylor et al. Figure S6

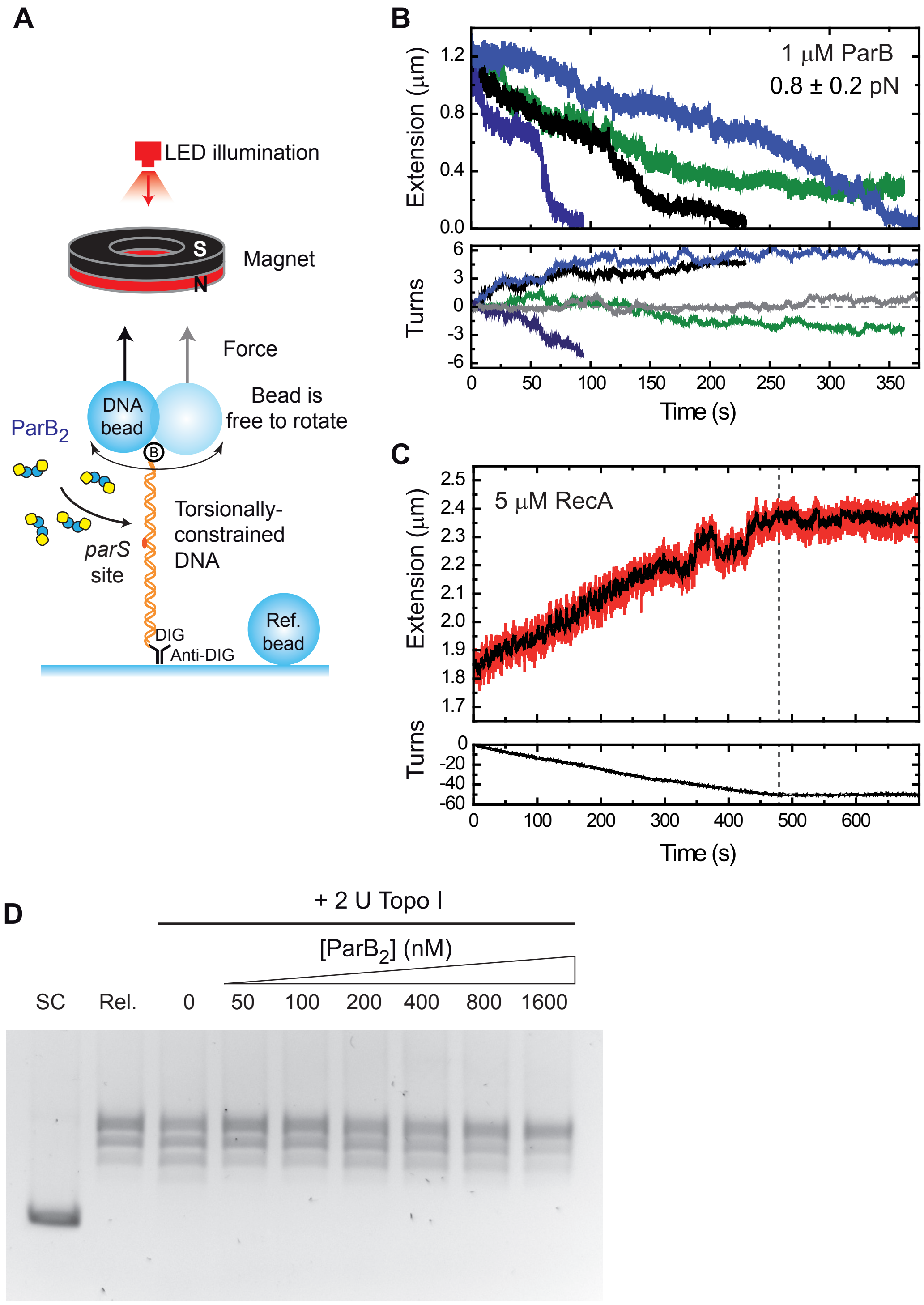

Taylor et al. Figure S7

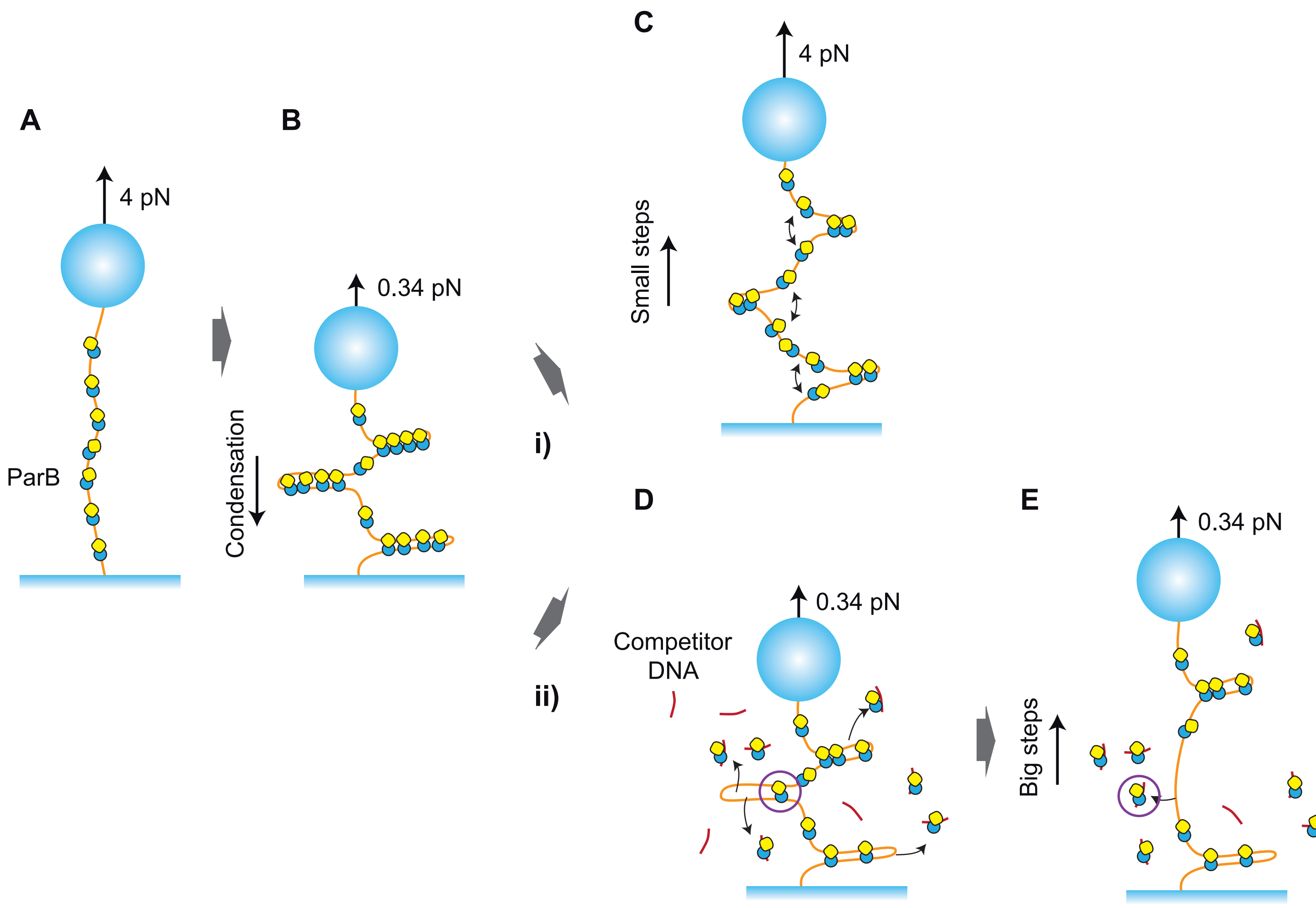

Supplement: SUPPLEMENTARY DATA [file supp_gku1295_nar-02970-f-2014-File011.pdf]
